# Guidelines for Antibiotic Prescription in Intensive Care Unit

**DOI:** 10.5005/jp-journals-10071-23101

**Published:** 2019-01

**Authors:** GC Khilnani,, Kapil Zirpe,, Vijay Hadda,, Yatin Mehta,, Karan Madan,, Atul Kulkarni,, Anant Mohan,, Subhal Dixit,, Randeep Guleria,, Pradeep Bhattacharya,

**Affiliations:** 1,3,5,7,9 Department of Pulmonary Medicine and Sleep Disorders, All India Institute of Medical Sciences, New Delhi, India,; 2 Neuro-Trauma Unit, Grant Medical Foundation, Ruby Hall Clinic, Pune, Maharashtra, India,; 4 Indian Society of Critical Care Medicine, Medanta Institute of Critical Care and Anesthesiology, Gurugram, Haryana, India,; 6 Department of Anaesthesiology, Division of Critical Care Medicine, Critical Care and Pain, Tata Memorial Hospital, Mumbai, Maharashtra, India,; 8 Sanjeevan and MJM Hospital, Pune, Maharashtra, India,; 10 Department of Anaesthesiology, Critical Care and Emergency Services, Bhopal, Madhya Pradesh, India

## Abstract

**How to cite this article:** Khilnani GC, Zirpe K, Hadda V, Mehta Y, Madan K, Kulkarni A, Mohan A, Dixit S, Guleria R, Bhattacharya P. Guidelines for Antibiotic Prescription in Intensive Care Unit. Indian Journal of Critical Care Medicine 2019;23 (Suppl 1):1-63.

## EXECUTIVE SUMMARY

### Pharmacokinetics and Pharmacodynamics

#### Evidence Statement

Time-dependent antibiotics require drug concentrations greater than the minimum inhibitory concentration (MIC) for a certain period between doses, which usually ranges from 40 to 50% of the inter-dose interval for their best action. Continuous infusions are preferred over extended infusions for beta-lactam antibiotics and are associated with clinical benefits like a decrease in hospital stay, cost of therapy and mortality. For vancomycin, continuous infusion is associated with reduced toxicity and cost of therapy but no mortality benefit.

## COMMUNITY-ACQUIRED PNEUMONIA IN THE INTENSIVE CARE UNIT

### What are the Common Organisms Causing Community-acquired Pneumonia in Intensive Care Unit Worldwide and India?

#### Evidence Statement

*Streptococcus pneumoniae*, gram-negative bacilli (including *klebsiella, Haemophilus influenzae*), atypical organisms (*Mycoplasma pneumoniae*) and viruses (including influenza) are common causes of community-acquired pneumonia (CAP) in intensive care unit (ICU). *Staphylococcus aureus, Legionella*, and *Mycobacterium tuberculosis* are less common causes of CAP in ICU. *Pseudomonas aeruginosa* is an important pathogen causing CAP in patients with structural lung disease. Methicillin-resistant *Staphylococcus aureus* (MRSA) and multidrug-resistant gram-negative organisms are relatively infrequent causes of CAP in India and are associated with risk factors such as structural lung disease and previous antimicrobial intake. Anaerobic organisms may cause CAP or co-infection in patients with risk factors for aspiration like elderly, altered sensorium, dysphagia, head, and neck malignancy. *S. pneumoniae* remains sensitive to beta-lactams and macrolides. *Haemophilus influenzae* has good sensitivity to beta-lactam with beta-lactamase inhibitors and fluoroquinolones. Recent studies show an increasing prevalence of extended spectrum β-lactamase (ESBL) producing Enterobacteriaceae.

### What are the Risk Factors for Multidrug-Resistant (MDR) Pathogens for CAP in ICU?

#### Evidence Statement

Risk factors for multidrug-resistant (MDR) organisms include age > 65 years, antimicrobial therapy in the preceding 3 months, high frequency of antibiotic resistance in the community, hospitalization for ≥ 48 hours in the preceding 3 months, home infusion therapy including antibiotics, home wound care, chronic dialysis within 1 month, family member with MDR pathogen and ongoing immunosuppressive treatment.

#### Recommendations

- All patients admitted with CAP in ICU should be evaluated for risk factors for infection with MDR organisms (2A).
- Antibiotic therapy should be individualized to cover the commonly implicated organisms according to risk factors, including *Pseudomonas*, ESBL producing Enterobacteriaceae or MRSA (3A).

### How Early should the Antibiotics be Initiated in Patients with CAP Who Require ICU Admission?

#### Evidence Statement

Early initiation of antibiotics has been associated with a reduction in all-cause mortality in community-acquired pneumonia, including severe pneumonia with sepsis or septic shock.

#### Recommendations

- Appropriate antimicrobial therapy should be initiated as early as possible in patients of CAP requiring ICU admission, preferably within the first hour after obtaining necessary microbiologic samples (3A).

### Should CAP in ICU Receive Empirical Antimicrobials or Upfront Targeted Antimicrobial Therapy?

#### Evidence Statement

Early institution of targeted antibiotic therapy in severe CAP based on urinary antigen testing is associated with a higher relapse rate without any mortality benefit in prospective randomized studies. Retrospective studies have shown mortality benefit with narrowing down of antibiotic therapy based on results from cultures of respiratory specimens, blood cultures as well as *Legionella* and pneumococcal urinary antigen testing.

#### Recommendations

- Empirical therapy covering common etiologic organisms should be initiated for severe CAP requiring ICU admission (2A).
- Investigations including the culture of respiratory secretions (sputum, endotracheal aspirate), blood cultures, urinary antigen testing for *Pneumococcus* and *Legionella* may be performed to narrow down therapy. Bronchoscopic BAL or protected specimen brush samples or polymerase chain reaction (PCR) for viral etiology may be performed for microbiologic diagnosis on a case by case basis (3A).

### For Empirical Therapy in Patients with CAP in ICU, should Combination Therapy be Preferred over Monotherapy?

#### Evidence Statement

Empirical combination therapy covering common organisms causing community-acquired pneumonia improves survival without any significant increase in microbial resistance.

#### Recommendations

- Patients with CAP requiring ICU admission should initially receive a combination of empirical antimicrobial agents covering common causative organisms (2A).

### What should be the Preferred Combination Therapy for CAP in ICU?

#### Evidence Statement

For patients with severe CAP requiring ICU admission without risk factors for pseudomonal infection, a combination of beta-lactams along with macrolides is better as compared to beta-lactam fluoroquinolone combination in terms of mortality benefit and length of hospital stay.

#### Recommendations

- For patients with CAP requiring ICU admission, a non-pseudomonal beta-lactam (cefotaxime, ceftriaxone, or amoxicillin-clavulanic acid) plus a macrolide (azithromycin or clarithromycin) should be preferred if there are no risk factors for *Pseudomonas aeruginosa* infection (1A).
- For penicillin-allergic patients, a respiratory fluoroquinolone (levofloxacin, moxifloxacin or ciprofloxacin) and aztreonam may be used (3A).
- If macrolides cannot be used, a fluoroquinolone may be used if there is no clinical suspicion of *tuberculosis*, after sending sputum or endotracheal aspirate for AFB and Genexpert (3A).

### When should Anti-pseudomonal Cover be Added for CAP in ICU? If Required, Which are the Preferred Antimicrobials for Antipseudomonal Cover?

#### Evidence Statement

For patients with severe CAP requiring ICU admission, risk factors for infection with *Pseudomonas aeruginosa* include chronic pulmonary disease (chronic obstructive pulmonary disease, asthma, bronchiectasis), frequent systemic corticosteroid use, prior antibiotic therapy, old age, immunocompromised states, enteral tube feeding, cerebrovascular or cardiovascular disease. Prior antibiotic therapy is a risk factor for multidrug-resistant pseudomonal infection.

#### Recommendations

- If *P. aeruginosa* is an etiological consideration, antipneumococcal, antipseudomonal antibiotic (like ceftazidime, cefoperazone, piperacillin-tazobactam, cefoperazone–sulbactam, imipenem, meropenem or cefepime) should be used (2A).
- Combination therapy should be considered with the addition of aminoglycosides or antipseudomonal fluoroquinolones (e.g., ciprofloxacin) (3A).

### When should MRSA Cover be Added to the Empiric Regimen for CAP in ICU?

#### Evidence Statement

Risk factors for MRSA in CAP in ICU include close contact with MRSA carrier or patient, influenza, prisoners, professional athletes, army recruits, men having sex with men (MSM), intravenous (IV) drug abusers, regular sauna users and those with recent antibiotic use. MRSA pneumonia should be suspected after influenza or in previously healthy young patients, if there is cavitation or necrotizing pneumonia, along with rapid increase of pleural effusion, massive hemoptysis, neutropenia or erythematous rashes. Vancomycin, teicoplanin, linezolid, and tigecycline are effective antibiotics against MRSA.

#### Recommendations

- All patients admitted with CAP in ICU should be evaluated for the presence of risk factors associated with MRSA (3A).
- If MRSA is a consideration, empiric vancomycin (1A) or teicoplanin (2A) should be added to the regimen. Linezolid should be used for vancomycin intolerant patients, vancomycin-resistant *Staphylococcus aureus* (VRSA), or patients with renal failure (1A).

### When should Anaerobic Cover be Added to the Empiric Antibiotic Regimen for CAP in ICU?

#### Evidence Statement

Risk factors for aspiration pneumonia in patients admitted with CAP in ICU include dysphagia, altered sensorium, coma, witnessed aspiration, putrid discharge, the presence of lung abscess, empyema or necrotizing pneumonia.

#### Recommendations

- Empirical antibiotics with anaerobic coverage should be considered in the treatment of CAP in ICU in the presence of clinical risk factors for aspiration or presence of lung abscess, empyema or necrotizing pneumonia (2A).

### Which Antibiotic should be Preferred for Anaerobic Coverage for CAP in ICU?

#### Evidence Statement

Commonly prescribed empirical antibiotics for CAP in ICU such as ampicillin-sulbactam, amoxicillin-clavulanic acid, piperacillin-tazobactam, and carbapenems have excellent anaerobic coverage. Clindamycin and moxifloxacin are effective against aspiration and lung abscess caused by anaerobic organisms. Lung abscess and necrotizing pneumonia may require prolonged treatment up to 4 to 6 weeks.

#### Recommendations

- Patients with CAP at risk of anaerobic infection should be initiated on antibiotics with anaerobic activity such as amoxicillin-clavulanate, clindamycin or moxifloxacin (1A).
- Piperacillin-tazobactam or carbapenems can be used for empirical therapy in CAP due to anaerobes if otherwise indicated (3A).
- Duration of treatment should be individualized according to the response and severity of the disease (3A).

### What should be the Optimal Duration of Antibiotics for CAP in ICU?

#### Evidence Statement

For CAP in ICU, there is limited evidence regarding the duration of treatment, with no significant mortality benefit beyond 7 days of antimicrobial therapy in uncomplicated cases. However, CAP due to GNB, Enterobacteriaceae, *P. aeruginosa, S. aureus* bacteremia, and *L. pneumophila* requires prolonged treatment. Necrotizing pneumonia, lung abscess, empyema or extrapulmonary infective complications like meningitis or infective endocarditis also require a longer duration of treatment.

#### Recommendations

- Patients with CAP requiring ICU admission should receive antibiotics for 7 to 10 days (2A).
- Patients with CAP due to *Pseudomonas* or aspiration pneumonia should be treated for 14 days (3A).
- Necrotizing pneumonia due to GNB, MRSA or anaerobes also require treatment for 14 to 21 days (3A)
- Duration of treatment should be individualized according to causative organism, response, the severity of disease and complications (3A).

### Should Procalcitonin be Used to Determine the Duration of Antibiotic Administration for CAP in ICU?

#### Evidence Statement

Serial procalcitonin levels can be used for de-escalation of antibiotics for CAP in ICU, without any increase in mortality or recurrence rates.

#### Recommendations

- Procalcitonin levels can be used along with clinical judgment for de-escalation of antibiotics in CAP in ICU in patients treated beyond 5 to 7 days (1A).

## VENTILATOR-ASSOCIATED PNEUMONIA

### What are the Common Organisms Causing HAP/VAP in ICU and What is their Antibiotic Susceptibility Pattern?

#### Evidence Statement

Ventilator-associated pneumonia (VAP) and hospital-acquired pneumonia (HAP) are commonly caused by aerobic gram-negative bacilli, such as *Acinetobacter baumannii, Klebsiella pneumoniae, Pseudomonas aeruginosa*, or by gram-positive cocci (*Staphylococcus aureus*). In Indian ICUs, gram-negative organisms are the most common etiologic agents (i.e., *Acinetobacter, Klebsiella* and *Pseudomonas spp*). Most of these pathogens have been found to be multidrug resistant. The frequency of specific MDR pathogens causing HAP and VAP may vary by hospital, patient population, type of ICU patient, and change over time.

### What are the Risk Factors for MDR Pathogens in VAP in ICU?

#### Evidence Statement

The risk factors for VAP due to MDR organisms include age > 60 years, duration of mechanical ventilation ≥ 7 days, prior antibiotic use within 3 months, the presence of severe sepsis or septic shock at the time of VAP, ARDS preceding VAP, renal replacement therapy before VAP and systemic corticosteroid therapy.

### What should be the Initial Combination of Empiric Antibiotic Therapy for VAP in ICU?

#### Evidence Statement

Use of antibiotic monotherapy and combination therapy for VAP have similar outcomes in patients who are not at risk for MDR pathogens. Commonly used antimicrobial agents include piperacillin-tazobactam, cefepime, levofloxacin, imipenem, and meropenem. Among antimicrobial agents, carbapenems have a higher chance of clinical cure than non-carbapenems. For treatment of VAP due to MRSA, glycopeptides and linezolid have similar clinical success; however, linezolid may be associated with a higher chance of thrombocytopenia and gastrointestinal adverse events.

#### Recommendations

- Among patients with VAP who are not at high risk of MDR pathogens and are in ICUs with a low prevalence of MRSA (< 15%) and resistant gram-negative organisms (<10%), single antibiotic active against both MSSA and *Pseudomonas* is preferred over combination antibiotic (1A)
- Among patients with VAP who are at high risk of MDR pathogens or are in ICU with a high prevalence of MRSA (>15%) and resistant gram-negative organisms (>10%), an agent active against MRSA and at least two agents active against gram-negative organisms including *P. aeruginosa* is recommended (3A)
- Among patients with VAP who are not at high risk of MDR pathogens and are in ICU with a high prevalence of resistant gram-negative organisms (>15%) but low prevalence of MRSA (<10%), two agents active against gram-negative organism including *P. aeruginosa* is recommended (3A)
- Colistin is not recommended for routine use as an empirical agent in VAP. However, it may be used upfront in the ICUs if there is a high prevalence of carbapenem-resistant *Enterobacteriaceae* (> 20%) (UPP).
- In our country or areas with high endemicity of *tuberculosis*, use of linezolid may be restricted unless no suitable alternative is available (UPP).
- Fluoroquinolones and aminoglyosides should be cautiously used as monotherapy in VAP in our country as well as in other areas with high endemicity of *tuberculosis* (UPP).
- In ICUs where the distribution of pathogen and antibiotic resistance pattern is known, empiric treatment should be designed accordingly, based upon patient risk factors for MDR pathogens. (UPP)

### When to Give Antipseudomonal Drugs for VAP in ICU?

#### Evidence Statement

Prior use of antibiotics (most consistent association), prolonged duration of mechanical ventilation, and chronic obstructive pulmonary disease (COPD) have been identified as risk factors for MDR *P. aeruginosa* infection.

#### Recommendations

- Empiric treatment should be given to cover *Pseudomonas* if there are risk factors for MDR *Pseudomonas* infection (2A).
- In ICUs where gram-negative isolate resistance rate is low (<10% gram-negative isolate resistant to the agent being considered for monotherapy) and patients have no risk factors for antimicrobial resistance, one anti-pseudomonal antibiotic may be given (3A).
- In ICUs where gram-negative isolate resistance rate is high (>10% gram-negative isolate resistant to the agent being considered for monotherapy or not known), two antipseudomonal antibiotics from a different class to be given (3A).

### What should be the Duration of Antibiotic Treatment for HAP/VAP?

#### Evidence Statement

Short-course regimens for VAP are associated with significantly more antibiotic-free days without any significant difference in the duration of ICU or hospital stay, recurrence of VAP and mortality. Short-course regimens are associated with more recurrences in VAP due to non-fermenting gram-negative bacilli (NF-GNB).

#### Recommendations

- Short course (7-8 days) of antibiotic therapy should be used, in the case of VAP with good clinical response to therapy (1A).
- Longer duration (14 days) of antibiotic therapy should be considered, in case of VAP caused by NF-GNBs or is associated with severe immunodeficiency, structural lung disease (COPD, bronchiectasis, and interstitial lung disease), empyema, lung abscess, necrotizing pneumonia, and inappropriate initial antimicrobial therapy (3A).

### When should Anaerobic Cover be Added for VAP and Which is the Preferred Antimicrobial Agent?

#### Evidence Statement

The incidence of anaerobic bacteria as the causative agent of VAP is 2 to 7%. Risk factors for VAP due to anaerobes are altered consciousness, aspiration pneumonitis and high simplified acute physiology score (SAPS).

#### Recommendations

- Empirical antibiotic regimen for VAP should not include coverage for anaerobic organisms routinely (2A).
- In the presence of risk factors for VAP due to anaerobic pathogens, anaerobic antimicrobial coverage should be added in an empirical regimen (2B).
- In patients with risk factors for anaerobic organisms, clindamycin or metronidazole should be added to empirical antibiotics regimen for VAP, if it does not include carbapenems (meropenem or imipenem) or piperacillin-tazobactam in the ongoing empirical regimen (UPP).

### When to Give Atypical Cover for VAP and Which is the Preferred Agent?

#### Evidence Statement

The incidence of atypical bacteria as causative agents of VAP is low (5 to 7.5%). Risk factors for VAP due to *Legionella* are *Legionella* colonization in hospital water supply, prolonged use of corticosteroids, cytotoxic chemotherapy, elderly, chronic renal failure, previous antibiotic use, granulocytopenia, and poor Glasgow coma score.

#### Recommendations

- Empirical antibiotic regimen for VAP should not include coverage for atypical organisms routinely (2A).
- In the presence of risk factors for VAP due to atypical bacterial pathogens, atypical antimicrobial coverage should be added to the empirical regimen (2B).
- The preferred atypical coverage in combination antibiotics regimen is fluoroquinolones (levofloxacin or moxifloxacin) or macrolides (azithromycin or clarithromycin) (UPP).

### Can Serum Procalcitonin be Used for De-escalation of Antibiotic Therapy in VAP?

#### Evidence Statement

Use of procalcitonin to guide de-escalation of antibiotic treatment in patients with VAP is effective in reducing antibiotic exposure, without an increase in the risk of mortality or treatment failure.

#### Recommendations

- Serum procalcitonin may be used to guide the de-escalation of antibiotics in VAP when the anticipated duration of therapy is > 7 to 8 days (1B).
- Serum procalcitonin levels (together with clinical response) should be used for de-escalation of antibiotic therapy in VAP in specific clinical conditions (severely immunocompromised patients, drug-resistant pathogens-NF-GNB, initial inappropriate therapy) (3A).

### How to Approach a Patient of Non-responding VAP?

#### Evidence Statement

Re-evaluation at 48 to 72 hours after the initial diagnosis of VAP is the most suitable time. By then the results of the initial microbial investigation are usually available, and treatment modification can be done. Evaluation of treatment response for VAP should be on the basis of clinical, laboratory, radiograph and microbiological results. Factors associated with treatment failure in VAP includes host factors (advanced age, immunosuppressed, chronic lung disease, ventilator dependence), bacterial factors (drug-resistant pathogens, opportunistic pathogens), therapeutic factors (inappropriate antibiotics, delayed initiation of therapy, insufficient duration of therapy, suboptimal dosing, inadequate local concentration of drugs), complications of initial VAP episode (lung abscess, empyema), other non-pulmonary infections or non-infectious mimics of pneumonia.

#### Recommendations

- Non-responding VAP should be evaluated for non-infectious mimics of pneumonia, unsuspected or drug-resistant pathogens, extrapulmonary sites of infection, and complications of pneumonia or its therapy and diagnostic testing should be directed to whichever of these causes is likely (2A).

## CATHETER-RELATED BLOODSTREAM INFECTIONS (CRBSI)

### What is the Incidence of Catheter Colonization and CRBSI?

#### Evidence Statement

The global incidence of CC ranges from 1.4 to 19.4 % whereas CRBSI incidence ranges from 2.4 to 12.5 %. The incidence of CC is higher in Indian ICUs ranging from 18 % to as high as 59%, whereas the incidence of CRBSI is up to 16.1 per 1000 catheter days.

### What are the Risk Factors for CRBSI?

#### Evidence Statement

Longer indwelling catheter duration, immunosuppression, diabetes mellitus, sepsis at the time of insertion, multi-lumen catheters and APACHE >23 are important risk factors for CRBSI. APACHE at admission, renal failure, central venous catheterization, and steroid therapy are important risk factors for fungal CRBSI.

### What are the Common Organisms Causing CRBSI and their Antibiotic Susceptibility?

#### Evidence Statement

Coagulase-negative *staphylococci* (CONS), *S. aureus, Enterococcus*, and *Candida* species are the common organisms accounting for the majority of the CRBSIs. A large proportion of *Staphylococcus aureus* and CONS are methicillin resistant ranging from 11 to 87%. There is an increased incidence of CRBSI due to gram-negative organisms (most of which are ESBL producers) and *candida* especially the non-albicans *candida*.

### What is/are the Empiric Antibiotic(s) of Choice for CRBSI in ICU?

#### Evidence Statement

Vancomycin, teicoplanin, linezolid, and daptomycin are effective in the treatment of CRBSI due to MRSA and MR-CONS. Fourth-generation cephalosporins, carbapenems or beta-lactam/beta-lactamase combination like piperacillin/tazobactam and aminoglycosides might be used for gram-negative organisms causing CRBSI. Caspofungin and fluconazole are equally effective as amphotericin-B for treatment of candidemia.

#### Recommendations

- Empirical antibiotic regimen for CRBSI should include coverage for both gram-positive and gram-negative organisms (2A).
- Vancomycin or teicoplanin is the recommended first-line drug for the empiric treatment of CRBSI for MRSA and MR-CONS while linezolid and daptomycin are good alternative agents (2A).
- Empiric coverage for gram-negative bacilli should include a fourth-generation cephalosporin, a carbapenem, or a β-lactam/β-lactamase inhibitor combination, with or without an aminoglycoside (UPP).
- An echinocandin or fluconazole should be used as empirical antifungal agents for the treatment of suspected central line-associated candidemia (2A).

### What should be the Duration of Antibiotic Treatment for CRBSI?

#### Evidence Statement

Short duration (< 14 days) of antibiotics is as effective as longer duration (>14 days) for uncomplicated *Staphylococcus aureus* bacteremia. Complicated bacteremia due to *S. aureus* or those associated with endocarditis should receive longer duration. For gram-negative bacteremia, seven days of antibiotics are sufficient. In responding patient with uncomplicated CONS infection, 5 to 7 days therapy is considered optimum. Minimum 14 days treatment with antifungals is required for fungal CRBSI.

#### Recommendations

- Minimum 2 weeks antibiotics should be given for uncomplicated and 4 to 6 weeks for complicated *Staphylococcus aureus* CRBSI and infective endocarditis (2A).
- Minimum 7 days of antibiotics should be given for gram-negative CRBSI (2A).
- Five to seven days antibiotics are recommended for CONS bacteremia (3A).
- For suspected fungal CRBSI, antifungal therapy for at least 14 days is recommended (UPP).

## URINARY AND UROGENITAL SEPSIS IN ICU

### What is the Incidence of UTI in ICU? What are the Common Organisms and Risk Factors for UTI in ICU?

#### Evidence Statement

The incidence of CA-UTI ranges from 5–30% of all ICU admissions. The most common organism causing UTI in ICU are gram-negative bacteria (*E. coli, Klebsiella*) and fungi (especially *Candida*). Risk factors for UTI in ICU include the duration of catheterization, length of ICU stay, prior antibiotic use, higher disease severity score, and female gender.

### What is the Empirical Antimicrobial Agent of Choice for Treating UTI in ICU?

#### Evidence Statement

There has been a trend towards increasing prevalence of extended-spectrum beta-lactamase producing gram-negative bacteria in the urinary cultures of catheter-associated UTI. Aminoglycosides, beta-lactams along with a beta-lactamase inhibitor as well as carbapenems and fosfomycin have good efficacy in catheter-associated UTI. The susceptibility for fluoroquinolones is decreasing over time among organisms isolated from nosocomial UTI. *Candida* species isolated from the patients with UTI show sensitivity to fluconazole.

#### Recommendations

- The initial choice of antibiotics should cover for ESBL producing gram-negative organisms and includes aminoglycosides, beta-lactam along with a beta-lactamase inhibitor or carbapenems (2A).
- In the initial empirical regimen for UTI, antibiotics against gram-positive organisms are not recommended (3A).
- In appropriate clinical settings, antifungals should be considered in the empirical regimen (3B).

## ACUTE INFECTIVE DIARRHEA, ANTIBIOTIC-INDUCED DIARRHEA, AND *CLOSTRIDIUM DIFFICILE*-ASSOCIATED DIARRHEA

### What are the Common Organisms Causing Acute Infective Diarrhea in the ICU?

#### Evidence Statement

The incidence of diarrhea in the ICU ranges from 12.9 to 38%. Majority of the cases of diarrhea in ICU are non-infectious in etiology. *Clostridium difficile* is responsible for the majority of infectious cases of diarrhea in ICU.

### What are the Empirical Antibiotics of Choice for Treating Acute Infective Diarrhea in the ICU?

#### Evidence Statement

Empirical use of metronidazole in patients with diarrhea suspected due to *Clostridium difficile* in ICU setting results in significant symptomatic improvement.

#### Recommendations

- We recommend that empiric metronidazole be used for therapy of patients with acute diarrhea in the ICU with suspected *Clostridium difficile* infection (3A).

### What are the Risk Factors for the Development of CDI or CDAD?

#### Evidence Statement

Risk factors for the development of CDI include prior antibiotic therapy, advanced age, prolonged ICU/hospital stay, immunosuppression, proton pump inhibitors, and enteral feeding. Cephalosporins, clindamycin, fluoroquinolones, carbapenems, and penicillin derivatives are the commonly implicated antibiotics for CDAD/CDI.

### What is the Recommended Treatment for CDI/CDAD: Which Antibiotics and Duration? Should Offending Antibiotics be Stopped? What is the Role of Probiotics in the Treatment of CDAD? How should Recurrent Clostridium difficile Infection be Treated?

#### Evidence Statement

Both metronidazole and oral vancomycin have similar efficacy in the clinical and bacteriologic cure of CDI. Use of implicated antibiotic after completing the treatment of CDI is associated with increased risk of recurrence of CDI. There is insufficient evidence to justify the use of probiotics as an adjunct to antibiotics in the treatment of CDAD. In a single RCT, fecal microbiota transplantation was found to be highly efficacious for treatment of recurrent CDI.

#### Recommendations

- We recommend metronidazole as the first line treatment of mild to moderate CDI/CDAD (1A).
- We recommend oral vancomycin as the first line treatment of microbiologically proven severe CDI/CDAD (1A).
- We recommend oral vancomycin as the treatment of recurrent CDI/CDAD infection (2A).
- We recommend fecal microbiota transplantation as an alternate treatment of recurrent CDI/CDAD infection (2A).
- We recommend that implicated antibiotics should be discontinued as soon as clinically feasible (2A).
- We recommend against the use of probiotics as an adjunct for the treatment of CDI/CDAD (2A).
- We recommend the addition of vancomycin to a patient with microbiologically proven CDI/CDAD if the patient is already on metronidazole or has no clinical response to metronidazole within 3 to 4 days (UPP)

## ABDOMINAL INFECTIONS IN ICU

### Acute Pancreatitis and Infected Pancreatic Necrosis

### What is the Incidence, risk factors, and microbiology of pancreatic infection following acute pancreatitis?

#### Evidence Statement

The incidence of pancreatic infection following acute pancreatitis ranges from 12 to 37%. Presence of pancreatic necrosis of > 50% is a major risk factor for pancreatic infection following acute pancreatitis. Primary organ failure predicts the development of infective pancreatic infection in patients with acute pancreatitis.

Gram-negative organisms are the most common organisms isolated from infected pancreatic necrosis following acute pancreatitis in Indian patients. Prophylactic antibiotic use in patients of AP to prevent IPN has been associated with increased risk of infection with gram-positive organisms. Resistance to carbapenems, beta-lactam/beta-lactamase inhibitors and quinolones in gram-negative organisms isolated from IPN has increased, however, with maintaining sensitivity to colistin and tigecycline.

### What are the Empirical Antibiotics of Choice for Treatment of Pancreatic Infection Following Acute Pancreatitis?

#### Evidence Statement

Prophylactic use of antibiotics in patients with necrotizing pancreatitis has not been shown to reduce the incidence of pancreatic infection and mortality. Presence of persistent fever, leucocytosis, multiorgan failure and presence of air within pancreatic necrosis suggest infected pancreatic necrosis. Cephalosporins, piperacillin-tazobactam, quinolones, and carbapenems have the highest whereas aminoglycosides have the lowest penetration into necrotic pancreatic tissue. Response to antibiotic therapy is assessed by clinical and radiological parameters.

#### Recommendations

- Routine use of prophylactic antibiotics to prevent pancreatic infection following acute pancreatitis of any severity is not recommended (1A)
- Empirical antibiotic regimen in patients with infected pancreatic necrosis should be guided by local microbiological data, susceptibility pattern, the pharmacokinetic property of antibiotics and previous antibiotic exposure (UPP).
- In treatment-naïve patients with evidence of infected pancreatic necrosis, we recommend empirical treatment with either carbapenems, piperacillin-tazobactam or cefoperazone- sulbactam (2A).
- In patients not responding or already exposed to the piperacillin-tazobactam, cefoperazone- sulbactam or carbapenems, colistin should be added to the empirical regime. (3B)
- Duration of antibiotic therapy should be guided by clinical, radiological and laboratory parameters (UPP).
- Patients not responding to antibiotics should undergo necrosectomy and drainage (3B).

## BILIARY SEPSIS

### Acute Cholangitis

### What is the Incidence, Risk Factors and Microbiology of Biliary Infection in ICU? What are the Empirical Antibiotics of Choice for Treatment of Biliary Infections in ICU?

### Incidence and risk factors

#### Evidence Statement

The incidence of acute cholangitis varies with underlying etiology and ranges from 0.2 to 10%. Cholelithiasis, choledocholithiasis, benign and malignant common bile duct (CBD) strictures, CBD interventions and stenting are the most common risk factors for cholangitis.

### Microbiology of Acute Cholangitis

#### Evidence Statement

Gram-negative organisms are the most common organisms isolated from patients with acute cholangitis. Most of the pathogens isolated are susceptible to third-generation cephalosporins (such as cefoperazone-sulbactam), aminoglycosides, quinolones, ureidopenicillins, and carbapenems. Risk factors for multidrug drug resistant organisms causing acute cholangitis include an indwelling biliary stent, malignant biliary obstruction, previous hospitalization and antibiotic use within 90 days.

### What is the Empirical Antibiotic Regimen for Acute Cholangitis?

#### Evidence Statement

The empirical antibiotic regime in patients with acute cholangitis is guided by the severity of the disease, local antibiotic susceptibility pattern and biliary penetration of the antibiotics. Duration of antibiotics depends on the severity of cholangitis and adequacy of source control. Biliary drainage (percutaneous or endoscopic) is required in addition to antibiotic use in the management of acute cholangitis.

#### Recommendations

- Empirical antibiotic therapy should be guided by the severity of the cholangitis, local microbiological susceptibility patterns, biliary penetration of antibiotics and previous antibiotic exposure (UPP).
- We recommend either beta-lactam/beta-lactamase inhibitor (such as cefoperazone-sulbactam or piperacillin/tazobactam) or carbapenems (imipenem/meropenem) as monotherapy in patients with moderate to severe cholangitis (3B).
- We recommend antibiotic duration for 4–7 days in patients of acute cholangitis after adequate source control (2B).
- Biliary drainage should be considered in all patients with cholangitis in addition to empirical antibiotic therapy (1A).

## LIVER ABSCESS

### Incidence and Risk Factors

### What are the most common organisms causing a liver abscess in ICU?

#### Evidence Statement

Amebic liver abscess is the most common cause of liver abscess in Indian setup. The incidence of pyogenic liver abscess varies from 2.3 to 446 per 100000 hospital admissions per year. Gram-negative organisms (*E. coli* and *Klebsiella*) are the most common organisms causing a pyogenic liver abscess. Risk factors for pyogenic liver abscess include diabetes mellitus, older age, male gender, biliary diseases, biliary procedures, alcoholism, malignancy, intra-abdominal infection, and cystic lesions in the liver.

### What are the Empirical Antibiotics of Choice for Treating a liver Abscess in ICU?

### Amebic Liver Abscess

#### Evidence Statement

Metronidazole is the drug of choice for treatment of amebic liver abscess. The optimum duration of treatment in patients with an amebic liver abscess is 10–14 days. Routine needle aspiration of amoebic liver abscess is controversial. Addition of aspiration to drug therapy in patients with amebic liver abscess of > 5 cm in size hastens clinical improvement.

#### Recommendations

- We recommend metronidazole as an initial antibiotic of choice in patients with an amoebic liver abscess (2A).
- We recommend antibiotic treatment for 10–14 days in patients with an amoebic liver abscess (3B).
- Needle aspiration of amebic liver abscess is recommended in patients with lack of clinical improvement in 48 to 72 hours, left lobe abscess, abscess more than 5 to 10 cm or thin rim of liver tissue around the abscess (< 10 mm) (UPP).

### Pyogenic Liver Abscess

#### Evidence Statement

Beta-lactam/beta-lactamase inhibitors, metronidazole, and carbapenems are effective antibiotics for the management of pyogenic liver abscess. Carbapenems are effective in case of suspected infection with ESBL producing organisms or melioidosis. Antibiotics are required for prolonged periods ranging from 2 to 4 weeks. Clinical and radiological assessment is required to guide the adequate treatment duration.

#### Recommendations

- We recommend beta-lactam/beta-lactamase inhibitors with metronidazole in patients with pyogenic liver abscess for a duration of 2–4 weeks (2A).
- We recommend carbapenems in case of infection with ESBL producing organisms or melioidosis (2B).

## PERITONITIS

### What are the Most Common Organisms Causing Peritonitis in ICU?

#### Evidence Statement

The risk factors for the development of primary peritonitis are decompensated cirrhosis, nephrotic syndrome and peritoneal dialysis. The risk factors for the development of secondary peritonitis include intra-abdominal organ perforation, post-intra-abdominal surgery, and trauma. Longer ICU stay, urgent operation on hospital admission, total parenteral nutrition, and stomach-duodenum as primary infection site are associated with the development of tertiary peritonitis. Gram-negative enteric organisms are the common causes of primary and secondary peritonitis. Other organisms include gram-positive as well as anaerobic bacteria. The organisms commonly isolated in tertiary peritonitis are *Candida, Enterococcus faecium* and *Staphylococcus epidermidis*.

### What are the Empirical Antibiotics of Choice for Treating Peritonitis in ICU?

#### Evidence Statement

Third generation cephalosporins are the most effective antibiotic therapy for primary peritonitis. Antibiotics are usually required for 7 to 10 days for adequate treatment. Most of the organisms isolated in secondary peritonitis are sensitive to beta-lactam/beta-lactamase inhibitors or carbapenems. For gram-positive organisms, vancomycin and linezolid are effective treatment options. The short duration of antibiotic treatment (4 days) is as effective as longer duration after an adequate source control.

#### Recommendations

- We recommend third-generation cephalosporins (such as cefotaxime and ceftriaxone) for 7 to 10 days in patients with primary peritonitis (2A).
- We recommend either the beta-lactam/beta-lactamase inhibitor or carbapenems with an anaerobic cover (using metronidazole) for the treatment of secondary peritonitis (2A).
- For secondary peritonitis antibiotic treatment is required for 4 days after an adequate source control (2A).

## CNS INFECTIONS IN ICU

### What are the Most Common Organisms Causing Acute Bacterial Meningitis in ICU?

### Community-acquired Meningitis

#### Evidence Statement

The incidence of community-acquired pyogenic meningitis ranges from 2 to 7.40 per lakh population. The common causative organisms include *Streptococcus pneumoniae, Neisseria meningitides*, other *streptococci, Haemophilus influenzae*, and *Listeria monocytogenes*. Other causative organisms are *Staphylococcus* species, gram-negative bacilli, *Pseudomonas*, and *Acinetobacter*. Common risk factors for community-acquired bacterial meningitis are otitis media, elderly population, depressed immune status and prior use of antibiotics.

### Nosocomial Meningitis

#### Evidence Statement

The incidence of post ventricular drain or catheter meningitis ranges from 2 to 27%. Commonly implicated organisms are CONS (especially *Staphylococcus epidermidis*), *Staphylococcus aureus, Acinetobacter, Pseudomonas*, and *Enterobacteriaceae*. Risk factors are repeated catheterization, higher catheter duration, CSF sampling, the presence of concomitant systemic infection and surgical technique, i.e., subcutaneously tunneled extraventricular drain (EVD), Rickham reservoir with percutaneous CSF drainage. The incidence of post craniotomy or post neurosurgery meningitis is 0.02% to 9.5%. Most commonly implicated organisms are *Staphylococcus aureus*, coagulase-negative *staphylococci* (especially *S. epidermidis*), *Enterobacteriaceae, Acinetobacter* and *Pseudomonas*. Risk factors include CSF leak, EVD, longer duration of drainage, multiple operations, lack of antibiotic prophylaxis and emergency surgery. The incidence of post-neuroaxial blockade meningitis is 0.2 per 10000 with viridans *streptococci* and *Staphylococcus aureus* being common organisms. Exogenous inoculation is the main risk factor. Post head trauma meningitis incidence ranges from 1.39% to 2% with CONS, and *Enterobacteriaceae* as common microbes and prolonged hospitalization, insertion of the lumbar and ventricular drain as common risk factors. Post internal ventricular drain infection incidence ranges from 5.9 to 15.2%. Most common causative organisms are CONS, *Staphylococcus aureus*, gram-negative bacilli, group D *streptococci*, and *Propionibacterium acnes*. CSF leak, single gloves use and a number of times shunt exposed to breached surgical gloves are the risk factors.

### What are the Empirical Antibiotics of Choice for Treating Acute Bacterial Meningitis in ICU? What Should be the Duration of Antibiotic Treatment?

### Community-acquired Meningitis

#### Evidence Statement

Choice of antibiotics depends on the most likely causative micro-organism, local antibiotics sensitivity patterns, mechanism of infection and patient's predisposing condition. Most commonly recommended empirical antibiotic regimens include third-generation cephalosporin plus vancomycin, third-generation cephalosporin monotherapy and penicillin monotherapy. Addition of amoxicillin, ampicillin or benzyl-penicillin has been recommended in patients older than 50 years.

#### Recommendations

- We recommend third-generation cephalosporin (preferably ceftriaxone) plus vancomycin as empirical antibiotics of choice for community-acquired meningitis (3A).
- We recommend adding ampicillin or amoxicillin if age > 50 years. (3A).
- If beta-lactams are contraindicated, we recommend chloramphenicol plus vancomycin as antibiotic of choice, and to add cotrimoxazole, if age > 50 years (3A).
- We recommend ciprofloxacin or aztreonam plus vancomycin as alternative regimen and to add cotrimoxazole if age greater than 50 years (UPP).
- We recommend a duration of antibiotics based on suspected or isolated organisms, i.e., 10 to 14 days for *Streptococcus pneumoniae*, 14 to 21 days for *Streptococcus* agalactiae, 7 days for *Neisseria meningitides* or *Haemophilus influenzae*, 21 days for aerobic gram-negative bacilli, and 21 days or more for *Listeria monocytogenes* (3A).
- If no microorganism is identified, treatment should be given for at least 10 to 14 days (3A).

### Nosocomial Meningitis

#### Evidence Statement

Vancomycin in combination with cefepime, ceftazidime or meropenem is commonly recommended an empirical antibiotic regimen for nosocomial meningitis. Alternative regimens include third-generation cephalosporin or meropenem monotherapy or ceftriaxone plus flucloxacillin or cloxacillin combination therapy. Limited available evidence shows the efficacy of intraventricular or intrathecal antibiotics in the management of nosocomial meningitis poorly responsive to systemic antibiotics.

#### Recommendations

- We recommend vancomycin plus cefepime or ceftazidime or meropenem as empirical antibiotics of choice for nosocomial meningitis (3A).
- Colistin may be given if the incidence of CRE or drug-resistant *Acinetobacter* is high in the specific unit (UPP).
- If beta-lactams are contraindicated, we recommend replacing β-lactam with aztreonam or ciprofloxacin (3A).
- Intraventricular/intrathecal antibiotics should be considered if infection responds poorly to appropriate systemic antibiotics clinically or microbiologically (3A).

### What are the Most Common Organisms Causing Brain Abscess in ICU?

#### Evidence Statement

The incidence of brain abscess ranges from 1.3 to 2.6 cases per lakh population. Most commonly involved micro-organism*s* include *Streptococcus* (especially *S. viridans*), *Staphylococcus* (especially *S. aureus*), gram-negative bacilli, anaerobes (*Bacteroides, Peptostreptococcus, Fusobacterium*), *Pseudomonas* and *H. influenzae*. Polymicrobial etiology accounts for 23–26% cases. Risk factors include otitis media, sinusitis, head trauma, congenital heart diseases, hematogenous spread, surgery, immunocompromised status, pulmonary disease, meningitis, and odontogenic infections.

### What are the Empirical Antibiotics of Choice for Treating Brain Abscess in ICU? What should be the Duration of Antibiotic Treatment?

#### Evidence Statement

The most common empiric treatment consists of a third-generation cephalosporin combined with metronidazole. Antibiotic duration ranges from 4 to 8 weeks.

#### Recommendations

- We recommend 3rd generation cephalosporins plus metronidazole as the empirical antibiotic of choice for brain abscess (3A).
- We recommend adding vancomycin if a high likelihood of MRSA (3A).
- We recommend vancomycin plus ciprofloxacin if beta-lactams are contraindicated (3A).
- We recommend aztreonam if ciprofloxacin cannot be given or contraindicated (UPP).
- We recommend a minimum 4 weeks of therapy; however, duration may be extended according to clinic-radiological response irrespective of aspiration or excision of the abscess (3A).

## SKIN AND SOFT-TISSUE INFECTIONS (SSTI) IN ICU

### What are the Most Common Organisms and Risk Factors for SSTI in ICU?

#### Evidence Statement

Older age, diabetes mellitus, obesity, malignancy, cirrhosis, and longer ICU stay are risk factors for SSTIs. Gram-positive organisms (*Staphylococcus aureus*) are the most common organism responsible for the SSTIs. *E. coli* and *Pseudomonas* are common pathogens among gram-negative organisms. MRSA and ESBL producing gram-negative organisms are the most common causative agents for SSTIs in ICU. Monomicrobial necrotizing fasciitis is commonly caused by *Streptococcus pyogenes*; mixed coliforms, anaerobes, and *staphylococci* are common causes of polymicrobial necrotizing fasciitis.

### What are the Empirical Antibiotics of Choice for Treating SSTI in ICU? For Empirical Therapy, Should Combination Therapy be Preferred over Monotherapy?

#### Evidence Statement

Vancomycin, teicoplanin, daptomycin, and linezolid are effective in SSTIs caused by MRSA. Piperacillin-tazobactam and carbapenems are the most effective antibiotics for ESBL producing gram-negative organisms. Penicillin plus clindamycin are most effective antibiotics in monomicrobial necrotizing fasciitis, whereas a combination of piperacillin-tazobactam, fluoroquinolone and clindamycin are effective for polymicrobial necrotizing fasciitis.

#### Recommendations

- For moderate nonpurulent SSTI, we recommend intravenous penicillin or clindamycin as the first choice of antibiotics (2A).
- Severe nonpurulent SSTI should be treated with a combination of piperacillin-tazobactam along with coverage for MRSA (vancomycin, teicoplanin, daptomycin or linezolid) (2A).
- Concomitant surgical inspection or debridement should be considered for severe non-purulent SSTIs (2A).
- For severe purulent SSTI, incision and drainage followed by empiric antibiotics including piperacillin-tazobactam, along with MRSA coverage (vancomycin, teicoplanin, daptomycin or linezolid) is recommended (3A).
- Penicillin plus clindamycin is recommended for monomicrobial necrotizing infection caused by *Streptococcus pyogenes* or clostridial species. For polymicrobial necrotizing fasciitis, a combination of piperacillin-tazobactam, fluoroquinolone, and clindamycin is recommended (3A).

### What Should be the Duration of Antibiotic Treatment for SSTI?

#### Evidence Statement

A shorter course of antibiotic therapy is adequate for uncomplicated SSTIs while complicated SSTIs require a longer duration of antibiotic therapy.

#### Recommendations

- Severe nonpurulent SSTIs should be treated with at least 5 days of antibiotics (3A).
- Severe SSTIs with organ dysfunction should be treated with a prolonged course of antibiotics of 2–3 weeks duration (3A).

## SEPSIS OF UNKNOWN CAUSE IN ICU

### What is the Empirical Treatment for Sepsis of Unknown Cause in ICU?

#### Evidence Statement

Empirical therapy with dual class (with different mechanisms of action) combination antimicrobial therapy for sepsis of unknown cause in ICU is associated with have better clinical outcomes. Empirical therapy with either piperacillin-tazobactam or carbapenems in combination with aminoglycoside or fluoroquinolone has been shown to give appropriate broad coverage leading to better clinical outcomes as compared to monotherapy.

#### Recommendations

- We recommend empirical antimicrobial therapy with a combination of ceftriaxone and doxycycline or macrolide for community-acquired sepsis of unknown origin in ICU (UPP).
- We recommend empirical antimicrobial therapy with a combination of beta-lactam/beta-lactamase inhibitor and a fluoroquinolone or aminoglycoside for nosocomial sepsis of unknown origin in ICU (UPP).
- Empiric therapy should attempt to provide antimicrobial activity against the most likely pathogens based upon clinical features along with local patterns of infection and resistance (UPP).
- Duration of therapy is 7–10 days, though longer courses may be appropriate in patients with slow response (3B).

## EMPIRICAL ANTIFUNGALS FOR NON-NEUTROPENIC PATIENTS IN ICU

### What are the Risk Factors for Invasive Fungal Infections in ICU?

#### Evidence Statement

Risk factors for invasive fungal infections in non-neutropenic patients in ICU are surgery, total parenteral nutrition, renal replacement therapy, cardiopulmonary bypass >120 minutes, diabetes mellitus, central venous catheters, urinary catheters, *Candida* colonisation with colonization index >0.5, use of broad-spectrum antibiotics, acute renal failure, mechanical ventilation > 3 days and APACHE II score >16.

### What is the Role of Empirical Antifungals in Non-neutropenic Patients in ICU?

#### Evidence Statement

Empirical antifungals for non-neutropenic patients in ICU routinely has not been associated with a decrease in mortality or hospital length of stay. Empirical antifungals in patients at high risk for invasive fungal infections in ICU has been shown to reduce the incidence of subsequent proven invasive fungal infections.

#### Recommendations

- We do not recommend the routine use of empirical antifungals in non-neutropenic patients in ICU (1A)
- Empirical antifungals may be considered in critically ill patients with a high risk of invasive fungal infections to reduce the incidence of subsequent invasive fungal infections (1B).

### What is the Antifungal Agent of Choice and Duration of Empirical Therapy in Non-neutropenic Patients in ICU?

#### Evidence Statement

Fluconazole and caspofungin are useful as empirical antifungal therapy in non-neutropenic ICU patients at high risk of Invasive fungal infection. In India, the rate of fluconazole resistance is up to 7%, especially in non-albicans *Candida* species.

#### Recommendations

- We recommend fluconazole or caspofungin as preferred empirical antifungal agents in non-neutropenic ICU patients at risk for invasive fungal infection (1A).
- Caspofungin may be preferred in areas with high prevalence of fluconazole resistance (1B).
- Micafungin or anidulafungin may be used as alternative agents (3A).
- Recommended duration of empirical antifungal therapy is 2 weeks. (3A)

### Antibiotic Stewardship

#### Evidence Statement

Antibiotic stewardship programs in hospitalized patients are associated with a reduction in a number of antibiotic days, duration of hospital stay and all-cause mortality.

#### Recommendations

- All hospitals should have an antibiotic stewardship program including the intensive care units (2A).

### What are the Essential Strategies of Antibiotic Stewardship in an ICU Setting?

#### Evidence Statement

Antibiotic stewardship requires a multidisciplinary approach with integration of infectious disease physician, a microbiologist with logistic and financial support from hospital administration. Both enablement and restrictive strategies are useful in improving adherence to antibiotic stewardship programs. Restrictive strategies give immediate results. Enablement practices are more resource intensive. Most studies have used a combination of both the methods and have shown additive effects. Providing feedback to the treating team improves adherence. A single RCT has shown that a restrictive strategy alone may cause a delay in the initiation of antibiotics.

#### Recommendations

- Prospective audit of antibiotic use and/or preauthorization (if feasible) along with feedback to the treating team is recommended as part of an antibiotic stewardship program (1A).

### What is the Role of Antibiotic Cycling, Intravenous to Oral switch and De-escalation in the ICU?

#### Evidence Statement

Antibiotic cycling in the intensive care unit has not been adequately studied in randomized controlled trials. Non-randomized studies show significant heterogeneity in terms of site of study, a method of cycling and confounders like simultaneous infection control measures being employed. Evidence of benefit of antibiotic cycling is lacking, with few studies demonstrating a reduction in colonization though mortality and length of hospital stay remain unchanged.

#### Recommendations

- Antibiotic cycling should not be used as a method of antibiotic stewardship program (2A).

### Scheduled Intravenous to Oral Switch

#### Evidence Statement

Early intravenous to the oral transition of antibiotics reduce hospital length of stay and cost of care. There is no increase in mortality or other adverse events when this is done after assessing which patients can be safely transitioned to oral therapy.

#### Recommendations

- Antibiotic stewardship programs should implement strategies to improve the timely transition from parenteral to oral antibiotic therapy (2A).

### De-escalation in Intensive Care Unit

#### Evidence Statement

Pooled results from observational studies in an ICU setting do not show any increase in mortality with antibiotic de-escalation while significantly reducing antibiotic exposure days and ICU length of stay.

#### Recommendations

- Antibiotic de-escalation in the ICU is recommended as part of an antibiotic stewardship program (2A).

### What is the Role of Procalcitonin in Antibiotic De-escalation ICU?

#### Evidence Statement

Implementation of antibiotic de-escalation algorithm based on serial procalcitonin measurements has been shown to reduce mortality, length of ICU stay, the total duration of antibiotic days and health care costs.

#### Recommendations

- Procalcitonin based algorithms may be used for antibiotic de-escalation (1A).

## INTRODUCTION

Severe infections are among the common indications requiring admission to intensive care units (ICU). All physicians, irrespective of the specialty, deal with such patients. For these patients, effective antibiotic therapy is life-saving. The resistance to currently available antibiotics is increasing over the last few years. Secondly, only a few new antibiotics have been marketed during the last few years and will be made available in the coming years. Therefore, the best way to preserve the efficacy of existing antibiotics remains the appropriate use of these drugs. One way to do this may be to increase awareness and develop guidelines for the prescription of antibiotics. There are international as well as Indian guidelines covering some of the common infections encountered in ICU. However, none of the existing guidelines have comprehensively addressed the issue of empirical antibiotic prescription in ICU as a whole. Therefore, these guidelines are the consensus of experts from all over the country based upon available scientific literature.

### Scope of Guidelines

The scope of these guidelines includes an antibiotic prescription for common bacterial infections for pneumonia (community acquired, hospital-acquired and ventilator-associated), bloodstream infections, abdominal infection (hepato-biliary, pancreatic, urogenital), central nervous system, skin- and soft- tissue infections in patients admitted in ICU. These guidelines are for immunocompetent patients. The antibiotic prescription for critically ill immunocompromised patients is dealt in part II of this supplement.

## METHODOLOGY

The guidelines for antibiotic prescription in intensive care unit were framed by the Department of Pulmonary Medicine and Sleep Disorders, All India Institute of Medical Sciences, New Delhi under the aegis of Indian Society of Critical Care Medicine. The committee included experts (list enclosed) from various realms dealing with ICU infections, i.e., Critical Care, Pulmonary Medicine, Gastroenterology, Neurology, Nephrology, and Microbiology. The experts were divided into five groups. Review of literature was performed by searching various electronic databases including Pubmed and Embase. Cross-references from articles and all major international guidelines on the topics were also reviewed.

The experts in each group exchanged and reviewed relevant literature, and the consensus was derived on the scope and questions that needed to be answered in the formulation of the guidelines. The final expert committee meeting was held over two days at the All India Institute of Medical Sciences, New Delhi. After detailed discussion, guidelines were framed after a thorough review of the literature.

Modified grade system was utilized to classify the quality of evidence and the strength of recommendations (Table 1). The draft document thus formulated was reviewed by all committee members; comments and suggestions were incorporated after discussion, and a final document was prepared. The final document was reviewed and accepted by all expert committee members.

**Table 1.** Criteria for level of evidence and grading of strength of recommendations used in formulation of current guidelines

***Quality of Evidence***	***Level***
Evidence from ≥1 good quality and well conducted randomized control trial(s) or meta-analysis of RCT's	1
Evidence from at least 1 RCT of moderate quality, or well-designed clinical trial without randomization; or from cohort or case-controlled studies.	2
Evidence from descriptive studies, or reports of expert committees, or opinion of respected authorities based on clinical experience	3
Not backed by sufficient evidence; however, a consensus reached by the working group, based on clinical experience and expertise	Useful Practice Point (UPP)
***Strength of Recommendations***	***Grade***
Strong Recommendations to do (or not to do) where the benefits clearly outweigh the risk (or vice versa) for most, if not all patients	A
Weak Recommendations, where benefits and risk are more closely balanced or are more uncertain	B

### Pharmacokinetics and Pharmacodynamics

Pharmacokinetics deals with the time course of drug absorption, distribution, metabolism, and excretion while pharmacodynamics involves the relationship between drug concentration and its effects including toxicity. Each antibiotic has its pharmacokinetic profile through each class of antibiotics has its class-specific properties as well. Each class of antimicrobials has a different pharmacodynamic profile based on different inhibitory characteristics on bacteria.

Individualized dosing regimens using known pharmacokinetics and pharmacodynamic characteristics are important to optimize patient outcomes and minimize antimicrobial resistance. Pharmacokinetic profiles change over time in critically ill patients, warranting periodic reconsideration of dosing regimens.

The factors determining metabolism and effects of an antibiotic include basic antibiotic characteristics such as lipophilic or hydrophilic, patient statuses such as volume status and end organ function and changes in pathophysiologic characteristics, i.e., systemic inflammation and hemodynamics. Hydrophilic antibiotics have a low volume of distribution, predominantly renal clearance and low intracellular penetration as compared to lipophilic antibiotics. Examples of hydrophilic antibiotics include beta-lactams, aminoglycosides, vancomycin, linezolid, and colistin while lipophilic antibiotics are fluoroquinolones, macrolides, clindamycin and tigecycline.^1^

The antibiotics can be broadly classified into those with concentration-dependent killing activity and those with time-dependent killing activity. The examples of former include aminoglycosides, fluoroquinolones, metronidazole, colistin, and clindamycin whereas that of latter include beta-lactams, linezolid, and tetracyclines.

Sepsis affects drug metabolism by various mechanisms. Being a hyperdynamic state it (pharmacologically or pathophysiologically enhanced) can increase creatinine clearance and hepatic perfusion thus increasing drug removal. At the same time, sepsis-induced organ-dysfunction can reduce metabolism and elimination of the active drug. Renal replacement therapies can increase clearance for some drugs like piperacillin-tazobactam and meropenem. The body has adaptive methods for increasing drug clearance during states of multiorgan failure. For example, gastrointestinal clearance of ciprofloxacin is increased in renal failure while biliary clearance of piperacillin increases in renal failure. Serum protein concentration also affects the antibiotic concentration. Significant changes in free fractions of the drug are only relevant for highly protein-bound drugs (>95%). Small changes in protein binding result in huge relative changes in free (unbound) drug. Changes in protein binding will affect both clearances as well as the volume of distribution. Most antibiotics have low protein binding (<90%) except ceftriaxone (95% bound to albumin), ertapenem, teicoplanin, aztreonam, and daptomycin.

An open-label RCT involving 140 patients with sepsis compared continuous infusion of beta-lactams with intermittent infusion and demonstrated higher clinical cure rates and higher ventilator-free days in continuous infusion group without any mortality difference between the two groups.^2^ Similar results have been found in other studies as well through a double-blind study by Dulhunty et al. did not find any difference in ICU-free days, 90-day survival and clinical cure between continuous infusion and intermittent infusion groups.^3^ An individual patient data meta-analysis found significantly lower hospital mortality rates with continuous infusion of beta-lactams as compared to intermittent infusion in patients with severe sepsis.^4^ Regarding vancomycin, a meta-analysis including 11 studies comparing continuous versus intermittent infusion found that patients treated with continuous infusion had a significantly lower incidence of nephrotoxicity without any difference in treatment failure and mortality.^5^

#### Evidence Statement

Time-dependent antibiotics require drug concentrations greater than the minimum inhibitory concentration (MIC) for a certain time period between doses, which usually ranges from 40 to 50% of the inter-dose interval for their best action. Continuous infusions are preferred overextended infusions for beta-lactam antibiotics and are associated with clinical benefits like a decrease in hospital stay, cost of therapy and mortality. For vancomycin, continuous infusion is associated with reduced toxicity and cost of therapy without any mortality benefit.

### Community-acquired Pneumonia in the Intensive Care Unit

Community-acquired pneumonia (CAP) refers to symptoms suggestive of acute lower respiratory tract illness (a cough with or without expectoration, dyspnea, pleuritic chest pain) along with systemic manifestations (fever, chills, rigors or severe malaise), clinicoradiologic evidence (like crepitations or bronchial breath sounds;lobar or patchy consolidation or interstitial infiltrates) and no other explanation for the illness.^6,7^ CAP can simply be defined as pneumonia which is not acquired in hospital or long-term care facility.^8^

### What are the Common Organisms Causing Community-acquired Pneumonia in Intensive Care Unit Worldwide and India?

Common organisms causing CAP requiring intensive care admission worldwide include *Streptococcus pneumoniae* (12–43%), *Hemophilus influenza* (0–12%), *Legionella pneumophila* (0–30%), *Staphylococcus aureus* (0–19%), gram-negative enteric bacilli (0–27%), *Mycoplasma pneumoniae* (0–7%), *Chlamydia* species (0-2%), *Coxiella burnetti* (0–2%), and viruses (0–17%) including influenza (0–9%).^9^ In a recent active population-based surveillance study, *Streptococcus pneumoniae, Staphylococcus aureus*, and *Enterobacteriaceae* were more commonly implicated in CAP requiring intensive care (p < 0.001).^10^ Methicillin resistant *Staphylococcus aureus* (MRSA) remains an infrequent but important cause of CAP in intensive care unit (ICU) settings; however, evidence regarding prevalence and risk factors is limited to few observational studies, case series and case reports.^11-14^

The literature on the epidemiology of CAP in India comes from hospital-based observational studies and surveillance data as the ICU specific studies are not available. *Streptococcus pneumoniae* (2–35.8%), *Mycoplasma pneumoniae* (3–24%), *Chlamydia pneumoniae* (6–18%), *Legionella spp*. (2–15%), *Mycobacterium tuberculosis* (0–5%), *Haemophilus influenzae* (0–15.4%), *Staphylococcus aureus* (2–13%), *Klebsiella pneumoniae* (3–25.5%), other gram negative bacilli (0–19%) are the common organisms implicated in CAP requiring hospitalization in India.^15-38^ High prevalence of *Staphylococcus aureus* (26.7%) and MRSA causing CAP (60.9% of *staphylococci*) has been reported in one Indian study.^16^

Increasing age, active smoking, chronic obstructive pulmonary disease (COPD) and diabetes mellitus appear to be significant risk factors for the development of severe CAP. Structural lung disease and COPD are risk factors for infection due to *Pseudomonas aeruginosa*.^6,39,40^

*Streptococcus pneumoniae* largely remains sensitive to amoxicillin-clavulanic acid and azithromycin with only a few studies reporting resistance to amoxicillin-clavulanic acid (20%), levofloxacin (20%) and azithromycin (13%).^6,24,25,35^ There is limited data on antibiotic sensitivity patterns of other microbes. *H. influenzae* also seems to be largely sensitive to amoxicillin clavulanic acid and azithromycin; in one study, 23% isolates were resistant to amoxicillin-clavulanic acid, 13% were resistant to azithromycin whereas only 6% were resistant to cefuroxime.^35^ Gram-negative bacilli (GNB) are usually sensitive to beta-lactams and fluoroquinolones.^33^ However, in recent studies, prevalence of extended spectrum β-lactamase (ESBL)organisms appears to be increasing with resistance to carbapenems (16.6%), piperacillin-tazobactam (39.5%), and cefoperazone-sulbactam (42%) reported in a recent prospective study.^35^

#### Evidence Statement

*Streptococcus pneumoniae*, gram-negative bacilli (including *Klebsiella, Hemophilus influenzae*), atypical organisms (*Mycoplasma pneumoniae*) and viruses (including influenza) are common causes of community-acquired *pneumonia* (CAP) in intensive care unit (ICU). *Staphylococcus aureus, Legionella*, and *Mycobacterium tuberculosis* are less common causes of CAP in ICU. *Pseudomonas aeruginosa* is an important pathogen causing CAP in patients with structural lung disease. Methicillin-resistant *Staphylococcus aureus* (MRSA) and multidrug-resistant gram-negative organisms are relatively infrequent causes of CAP in India and are associated with risk factors such as structural lung disease and previous antimicrobial intake. Anaerobic organisms may cause CAP or co-infection in patients with risk factors for aspiration like elderly, altered sensorium, dysphagia, head, and neck malignancy. *S. pneumoniae* remains sensitive to beta-lactams and macrolides. *Hemophilus influenzae* has good sensitivity to beta-lactam with beta-lactamase inhibitors and fluoroquinolones. Recent studies show an increasing prevalence of extended spectrum β-lactamase (ESBL) producing Enterobacteriaceae.

### What are the Risk Factors for Multidrug-resistant (MDR) Pathogens for CAP in ICU?

Age more than 65 years, chronic respiratory disease, and prior antibiotic treatment were associated with increased risk of CAP due to multidrug-resistant (MDR) pathogens in prospective observational studies.^41-44^ Other factors associated with increased risk of MDR CAP include prior hospitalization for more than 48 hours in the last 3 months, home infusion therapy and patients on renal replacement therapy. Immunosuppression was also considered to be a risk factor for CAP due to MDR organisms.^6^

#### Evidence Statement

Risk factors for multidrug-resistant (MDR) organisms include age > 65 years, antimicrobial therapy in the preceding 3 months, high frequency of antibiotic resistance in the community, hospitalization for ≥ 48 hours in the preceding 3 months, home infusion therapy including antibiotics, home wound care, chronic dialysis within 1 month, family member with MDR pathogen and ongoing immunosuppressive treatment.

#### Recommendations

- All patients admitted with CAP in ICU should be evaluated for risk factors for infection with MDR organisms (2A).
- Antibiotic therapy should be individualized to cover the commonly implicated organisms according to risk factors, including *Pseudomonas*, ESBL producing *Enterobacteriaceae* or MRSA (3A).

### How Early should the Antibiotics be Initiated in patients with CAP Who Require ICU Admission?

In retrospective studies on CAP, initiation of antibiotics within 4 hours of presentation has been associated with a reduction in all-cause mortality, regardless of severity [relative risk (RR) 0.24; 95% confidence interval (CI) 0.08–0.71].^45^ A systematic review of prospective studies also favored early administration of antibiotics; however, the confidence interval was wide (RR 0.82; 95% CI 0.54–1.24).^45^ A recent meta-analysis of retrospective studies also showed decreased all-cause mortality with early administration of antibiotics before 4 hours of hospital admission, especially in severe CAP with pneumonia severity index (PSI) IV to V (adjusted odds ratio, AOR 0.87; 95% CI 78–97). However, no significant benefit was shown in clinical stability at 48 hours (AOR 1.04; 95% CI 0.75–1.44), length of hospital stay (AOR 0.92; 95% CI 84-1.01%) or readmission after discharge (AOR 0.99; 95% CI 0.88–1.11%).^8^ However, all the included studies were retrospective or chart reviews, with low quality of evidence. There was no significant mortality benefit with the administration of antibiotics before one hour of recognition of severe sepsis or septic shock (pooled odds ratio 1.46, 95% CI 0.89–2.4) in a recent meta-analysis. Out of 18 eligible studies, 7 studies were excluded due to non-availability of data confounding the findings.^46^ In a recent retrospective study of 35,000 randomly selected in patients with sepsis, each hour delay in administration of antibiotics was associated with increased odds of in-hospital mortality in patients with sepsis (Odds ratio, OR 1.09; 95% CI 1.00–1.19; p = 0.046), severe sepsis (OR 1.07; 95% CI 1.01–1.24; p = 0.014) and septic shock (OR 1.14; 95% CI 1.06–1.23; p = 0.001).^47^

#### Evidence Statement

Early initiation of antibiotics has been associated with a reduction in all-cause mortality in community-acquired pneumonia, including severe pneumonia with sepsis or septic shock.

#### Recommendations

- Appropriate antimicrobial therapy should be initiated as early as possible in patients of CAP requiring ICU admission, preferably within the first hour after obtaining necessary microbiologic samples (3A).

### Should CAP in ICU Receive Empirical Antimicrobials or Upfront Targeted Antimicrobial Therapy?

Targeted antibiotic therapy based on *Legionella* and pneumococcal urinary antigen testing was associated with higher relapse rate without any significant differences in clinical failure, length of hospital stay or clinical failure in a randomized controlled trial in patients with severe CAP. However, the study was inadequately powered for outcomes as less than 50% of patients had PSI IV, and V CAP and only one patient required ICU admission.^48^ In another randomized controlled trial, targeted antibiotic therapy based on respiratory secretions cultures, blood cultures, paired serum samples (for *Mycoplasma, Chlamydia*, and *Coxiella*) and urinary antigens (for *Pneumococcus* and *Legionella*) was similar to empirical therapy in terms of clinical cure, length of hospital stay and late treatment failure or relapse. The study was inadequately powered for ICU patients, though it demonstrated significantly reduced mortality (45% *vs*. 91%; p = 0.02) with targeted therapy as compared to empirical therapy.^49^ Similarly, in a large retrospective study, targeted antibiotic therapy has been associated with reduced 30-day mortality (AOR 0.64, 95% CI 0.56–0.74) in CAP, severe CAP (AOR 0.70; 95% CI 0.54–0.91)and very severe CAP (AOR 0.51, 95% CI 0.40 to 0.64).^8,50^ Other retrospective studies have demonstrated the limited utility of diagnostic testing to influence prescription modification, clinical cure or failure though lower mortality is reported with targeted therapy (RR 0.37, 0.24–0.57).^8,51^ Obtaining blood cultures before initiating therapy was associated with a mortality benefit in a large retrospective study in 14069 patients with CAP requiring hospitalisation.^52^

#### Evidence Statement

Early institution of targeted antibiotic therapy in severe CAP based on urinary antigen testing is associated with higher relapse rate without any mortality benefit in prospective randomized studies. Retrospective studies have shown mortality benefit with narrowing down of antibiotic therapy based on results from cultures of respiratory specimens, blood cultures as well as *Legionella* and pneumococcal urinary antigen testing.

#### Recommendations

- Empirical therapy covering common etiologic organisms should be initiated for severe CAP requiring ICU admission (2A).
- Investigations including the culture of respiratory secretions (sputum, endotracheal aspirate), blood cultures, urinary antigen testing for *Pneumococcus* and *Legionella* may be performed to narrow down therapy. Bronchoscopic BAL or protected specimen brush samples or polymerase chain reaction (PCR) for viral etiology may be performed for microbiologic diagnosis on a case by case basis (3A).

### For Empirical Therapy in Patients with CAP in ICU, should Combination Therapy be Preferred Over Monotherapy?

In a recent meta-analysis of CAP patients including 28 observational studies, combination antimicrobial regimens including macrolides have been associated with significantly decreased mortality as compared to non-macrolides (RR 0.82; 95% CI, 0.70–0.97; p = 0.02), along with a trend towards mortality benefit favoring macrolides as compared to fluoroquinolones (RR 0.83; 95% CI, 0.67–1.03; p = 0.09).^53^ Combination therapy also resulted in better survival in patients with shock without any significant increase in microbial resistance.^54^In a matched case-control study of prospectively studied cohorts, combination therapy including macrolides was an independent predictor of survival (OR, 0.19; 95% CI, 0.07–0.51) in patients with pneumococcal CAP requiring ICU admission.^55^

#### Evidence Statement

Empirical combination therapy covering common organisms causing community-acquired pneumonia improves survival without any significant increase in microbial resistance.

#### Recommendations

- Patients with CAP requiring ICU admission should initially receive a combination of empirical antimicrobial agents covering common causative organisms (2A).

### What should be the Preferred Combination Therapy for CAP in ICU?

In a recent meta-analysis of 8 studies (1 randomized controlled trial and 7 observational studies), 2273 patients in beta-lactam macrolide arm were compared to 1600 patients in beta-lactam-fluoroquinolonearm; beta lactam-macrolide combination was associated with a lower overall mortality as compared to that of beta lactam-fluoroquinolone combination (OR, 0.68; 95% CI 0.49–0.94; p = 0.02) along with decreased length of hospital stay (mean difference, −3.05 days; 95% CI, −6.01 to −0.09; p = 0.04).^56^ Aztreonam and fluoroquinolones are effective alternatives to macrolides, however, with undue risk of masking and delaying diagnosis of tuberculosis.^57^ Aztreonam is effective alternative for patients with contraindication to beta lactams.

#### Evidence Statement

For patients with severe CAP requiring ICU admission without risk factors for pseudomonal infection, a combination of beta-lactams along with macrolides is better as compared to beta-lactam fluoroquinolone combination in terms of mortality benefit and length of hospital stay.

#### Recommendations

- For patients with CAP requiring ICU admission, a non-pseudomonalbeta-lactam (cefotaxime, ceftriaxone, or amoxicillin-clavulanic acid) plus a macrolide (azithromycin or clarithromycin) should be preferred if there are no risk factors for *Pseudomonas aeruginosa* infection (1A).
- For penicillin-allergic patients, a respiratory fluoroquinolone (levofloxacin, moxifloxacin or ciprofloxacin) and aztreonam may be used (3A).
- If macrolides cannot be used, a fluoroquinolone may be used if there is no clinical suspicion of tuberculosis, after sending sputum or endotracheal aspirate for AFB and Genexpert (3A).

### When should Anti-pseudomonal Cover be Added for CAP in ICU? If Required, Which are the Preferred Antimicrobials for Anti-pseudomonal Cover?

Age greater than 65 to 70 years, male gender, current smokers, chronic respiratory disease including chronic bronchitis, COPD, asthma or bronchiectasis, cerebrovascular disease, dementia, other chronic neurological disorders, cardiovascular diseases, cirrhosis, immunocompromised states, malignancy, current use of corticosteroids, enteral tube feeding, previous hospital admission, prior antibiotic therapy and severe pneumonia at presentation have been reported as risk factors for CAP due to *Pseudomonas aeruginosa* in various observational studies.^30,42,43,58-62^ Prior antibiotic therapy has been associated with increased risk of multidrug-resistant pseudomonal infection.^60^

#### Evidence Statement

For patients with severe CAP requiring ICU admission, risk factors for infection with *Pseudomonas aeruginosa* include chronic pulmonary disease (chronic obstructive pulmonary disease, asthma, bronchiectasis), frequent systemic corticosteroid use, prior antibiotic therapy, old age, immunocompromised states, enteral tube feeding, cerebrovascular or cardiovascular disease. Prior antibiotic therapy is a risk factor for multidrug-resistant pseudomonal infection.

#### Recommendations

- If *P. aeruginosa* is an etiological consideration, antipneumococcal, antipseudomonal antibiotic (like ceftazidime, cefoperazone, piperacillin-tazobactam, cefoperazone–sulbactam, imipenem, meropenemorcefepime) should be used. (2A)
- Combination therapy should be considered with the addition of aminoglycosides or antipseudomonal fluoroquinolones (e.g., ciprofloxacin) (3A).

### When should MRSA Cover be Added to the Empiric Regimen for CAP in ICU?

Evidence on CAP due to MRSA is limited, and mostly based on small prospective studies, case series or case reports.^11-14^ A systematic review (81 studies; 7 case series, 71 case reports, 3 observational studies) estimated incidence of MRSA CAP to be 0.51 to 0.64 cases per 100,000 population.^63^ MRSA CAP carries high mortality (up to 60%). Close contact with a MRSA carrier or patient, preceding influenza infection, prisoners, professional athletes, army recruits, men having sex with men (MSM), intravenous drug abusers, regular sauna users, immunocompromised status (HIV, acute leukemia, ongoing systemic corticosteroid therapy)and those using antibacterial agents before infection have an increased risk of MRSA CAP.^63,64^ Multilobar consolidation, necrotizing consolidation and empyema were also observed in greater proportion of patients with MRSA CAP.^13^ Considering multiple risk factors, relatively low frequency but high morbidity and mortality associated with MRSA CAP, the expert group decided to emphasize on thorough assessment of risk factors for MRSA CAP in ICU, while balancing the Recommendations to guard against blanket MRSA cover for all CAP cases getting admitted to ICU. The most effective antibiotics against MRSA are vancomycin and teicoplanin. Tigecycline is also effective against MRSA; linezolid has also been reported to be effective in MRSA and VRSA pneumonia.^8,65^

#### Evidence Statement

Risk factors for MRSA in CAP in ICU include close contact with MRSA carrier or patient, influenza, prisoners, professional athletes, army recruits, men having sex with men (MSM), intravenous (IV) drug abusers, regular sauna users and those with recent antibiotic use. MRSA pneumonia should be suspected after influenza or in previously healthy young patients, if there is cavitation or necrotizing pneumonia, along with the rapid increase of pleural effusion, massive hemoptysis, neutropenia or erythematous rashes. Vancomycin, teicoplanin, linezolid, and tigecycline are effective antibiotics against MRSA.

#### Recommendations

- All patients admitted with CAP in ICU should be evaluated for the presence of risk factors associated with MRSA (3A).
- If MRSA is a consideration, empiric vancomycin (1A) or teicoplanin (2A) should be added to the regimen. Linezolid should be used for vancomycin intolerant patients, vancomycin-resistant *Staphylococcus aureus* (VRSA), or patients with renal failure (1A).

### When should Anaerobic Cover be Added to the Empiric Antibiotic Regimen for CAP in ICU?

Anaerobic organisms were reported to cause the majority of pulmonary infections associated with lung abscesses (26–100%), aspiration pneumonia (62–100%) and empyema (9–76%) in observational studies.^66-74^ In a recent observational study of 64 patients with CAP, 15.6% of BAL samples had evidence of anaerobic infection on 16s RNA analysis.^75^ Witnessed aspiration, loss of consciousness due to drug or alcohol overdose, seizures with concomitant gingival disease and dysphagia have been considered as risk factors for anaerobic infection.^76^

#### Evidence Statement

Risk factors for aspiration pneumonia in patients admitted with CAP in ICU include dysphagia, altered sensorium, coma, witnessed aspiration, putrid discharge, the presence of lung abscess, empyema or necrotizing pneumonia.

#### Recommendations

- Empirical antibiotics with anaerobic coverage should be considered for treatment of CAP in ICU in the presence of clinical risk factors for aspiration or presence of lung abscess, empyema or necrotizing pneumonia. (2A)

### Which Antibiotic should be Preferred for Anaerobic Coverage for CAP in ICU?

Clindamycin was associated with significantly higher cure rates as compared to penicillin in randomized controlled trials in anaerobic lung infections.^71,77^ In a randomized prospective study of 100 patients with anaerobic lung infections, ampicillin-sulbactam, clindamycin and panipenem-betamiprom had similar clinical efficacy (p = 0.62) and similar duration of treatment (p = 0.35) whereas non-clindamycin group had higher frequency of appearance of MRSA (22.7% *vs*. 0%; p < 0.01).^78^ Ampicillin-sulbactam had similar clinical and bacteriologic response to clindamycin with or without cephalosporin in another prospective randomized multicenter study of 70 patients with anaerobic lung infections.^79^ Moxifloxacin demonstrated a similar clinical response to ampicillin-sulbactam in a prospective open-label randomized multicentric study involving 139 patients with aspiration pneumonia and lung abscess, along with the added advantage of once-daily dosing.^80^ Moxifloxacin was also shown to be superior to levofloxacin-metronidazole combination in terms of clinical cure at 7 weeks (76.7% *vs*. 51.7%; p <0.05) as well as similar bacteriologic cure (93.3% *vs*. 96.4%, p >0.05) without any significant difference in adverse drug reactions.^81^ Duration of treatment has been reported to be variable. Longer duration of treatment (3–6 weeks) is required in lung abscesses and empyema.^71,79,80^

#### Evidence Statement

Commonly prescribed empirical antibiotics for CAP in ICU such as ampicillin-sulbactam, amoxicillin-clavulanic acid, piperacillin-tazobactam, and carbapenems have excellent anaerobic coverage. Clindamycin and moxifloxacin are effective against aspiration pneumonia and lung abscess caused by anaerobic organisms. Lung abscess and necrotizing pneumonia may require prolonged treatment up to 4 to 6 weeks.

#### Recommendations

- Patients with CAP at risk of anaerobic infection should be initiated on antibiotics with anaerobic activity such as amoxicillin-clavulanate, clindamycin or moxifloxacin (1A).
- Piperacillin-tazobactam or carbapenems can be used for empirical therapy in CAP due to anaerobes if otherwise indicated (3A).
- Duration of treatment should be individualized according to the response and severity of the disease (3A).

### What should be the Optimal Duration of Antibiotics for CAP in ICU?

On post-hoc analysis of an RCT comparing levofloxacin treatment for 5–10 days, the subgroup with moderate to high severity CAP had similar clinical cure rates (RR 1.07; 95% CI 0.95 to 1.2).^8,82^ In another study on severe CAP, treatment for more than 7 days did not confer any mortality benefit.^83^ However, this study excluded ICU admission, complicated pneumonia, non-responding pneumonia or identification of organisms requiring prolonged treatment. Also, *Enterobacteriaceae, Pseudomonas, Legionella*, and *S. aureus* were associated with the requirement of prolonged treatment.

#### Evidence Statement

For CAP in ICU, there is limited evidence regarding the duration of treatment, with no significant mortality benefit beyond 7 days of antimicrobial therapy in uncomplicated cases. However, CAP due to GNB, *Enterobacteriaceae, P. aeruginosa, S. aureus* bacteremia and *L. pneumophila* requires prolonged treatment. Necrotizing pneumonia, lung abscess, empyema or extrapulmonary infective complications like meningitis or infective endocarditis also require a longer duration of treatment.

#### Recommendations

- Patients with CAP requiring ICU admission should receive antibiotics for 7–10 days (2A).
- Patients with CAP due to *Pseudomonas* or aspiration pneumonia should be treated for 14 days (3A).
- Necrotizing pneumonia due to GNB, MRSA or anaerobes also require treatment for 14–21 days (3A)
- Duration of treatment should be individualized according to causative organism, response, the severity of disease and complications (3A).

### Should Procalcitonin be Used to Determine the Duration of Antibiotic Administration for CAP in ICU?

In a recent meta-analysis of 26 trials involving 6708 patients, procalcitonin utilization for antibiotic discontinuation was associated with reduced mortality (adjusted OR 0.83, 95 CI 0.70 to 0.99, p = 0.037).^84^

#### Evidence Statement

Serial procalcitonin levels can be used for de-escalation of antibiotics for CAP in ICU, without any increase in mortality or recurrence rates.

#### Recommendations

- Procalcitonin levels can be used along with clinical judgment for de-escalation of antibiotics in CAP in ICU in patients treated beyond 5–7 days (1A).

### Ventilator-associated Pneumonia

Pneumonia is one of the commonest hospitals acquired infection. Hospital-acquired or nosocomial pneumonia (HAP) is defined as pneumonia that occurs 48 hours (or more) after admission and did not appear to be incubating at the time of admission. Ventilator-associated pneumonia (VAP) is HAP that develops more than 48–72 hours after endotracheal intubation. The previously used term health care-associated pneumonia (HCAP) is currently not in use.^85^ To provide a more uniform and consistent reporting of cases of ventilator-associated complications, Centre for Disease Control (CDC) has proposed the term ventilator-associated events which include ventilator-associated condition, infection-related ventilator-associated complication, probable VAP and possible VAP.^86^ The incidence of VAP varies among different ICUs and depends upon the definition used. In most ICUs, the incidence is around 10–20%.^85^ Endotracheal intubation compromises the natural barrier between oropharynx and trachea as well as facilitates entry of bacteria into the lungs.^87^ Supine position also facilitates the transfer of contaminated secretions leading to VAP.^88^ VAP is suspected in patients with new or progressive pulmonary infiltrates plus supportive clinical findings suggestive of infection. The diagnosis is made on clinicoradiological findings and is supported by isolation of microorganism from lower respiratory tract sample. Critically ill patients who develop VAP are two times more likely to die as compared with similar patients without VAP. VAP leads to significantly longer ICU length of stay and also incur additional hospital costs.^89^

### What are the Common Organisms Causing HAP/VAP in ICU and what is their Antibiotic Susceptibility Pattern?

The microorganisms implicated in the causation of VAP varies among ICUs. Studies conducted in Western countries demonstrated that majority of VAP episodes are caused by *Staphylococcus aureus* followed by *Pseudomonas aeruginosa*.^90^ In a retrospective review of 8474 cases of VAP reported to CDC, *Staphylococcus* accounted for 24.1% of cases followed by *Pseudomonas* (16.6%) and *Klebsiella* (10.1%).^91^

Studies from Asia show preponderance of gram-negative organisms as an etiologic agent of VAP. A prospective surveillance study from 73 hospitals in 10 Asian countries from 2008 to 2009 including 2554 cases with HAP or VAP found that *Pseudomonas* (15.6%) was most common causative organism followed by *Staphylococcus aureus* (15.5%), *Acinetobacter spp*. (13.6%) and *Klebsiella* *pneumoniae* (12%). Imipenem resistance of *Acinetobacter* and *P. aeruginosa* was 67.3% and 27.2% respectively. A large proportion of *Acinetobacter* (82%) and *P. aeruginosa* (42.8%) were multidrug resistants (MDR) while 51.1% and 4.9% were extensively drug-resistant (XDR), respectively. The prevalence of MRSA among *S. aureus* isolates was 82.1%.^92^ Similarly, another retrospective study from Thailand also found *A. baumannii* (53.4%) as most common isolate followed by *P. aeruginosa* (35.2%) and MRSA (15.1%).^93^

Multiple studies from Indian ICUs have also shown predominance of gram-negative bacilli (*Acinetobacter*, and *Klebsiella*) in VAP.^94-96^ These gram-negative bacilli are often multidrug resistant. A prospective study from Pondicherry showed an incidence of VAP to be 18% where *Pseudomonas* and *Acinetobacter* were common (21.3%) followed by *Staphylococcus* (14.9%).^97^ Another study from Karnataka found *A. baumannii* to be the commonest organism in both early and late onset VAP followed by *Pseudomonas*. All isolates of *Acinetobacter* were resistant to at least three antibiotics (i.e., MDR) and one isolate of *Acinetobacter* was pan-resistant.^98^ There has been also a rise in carbapenem resistance of *Acinetobacter*. A study done by Gurjar et al. showed that 75% of patients with VAP due to *Acinetobacter* were carbapenem resistant.^99^

#### Evidence Statement

Ventilator-associated pneumonia (VAP) and hospital-acquired pneumonia (HAP) are commonly caused by aerobic gram-negative bacilli, such as *Acinetobacter baumannii, Klebsiella pneumonia*e, *Pseudomonas aeruginosa*, or by gram-positive cocci (*Staphylococcus aureus*). In Indian ICUs, gram-negative organisms are the most common etiologic agents (i.e., *Acinetobacter*, and *Pseudomonas* spp). Most of these pathogens have been found to be multidrug resistant. Frequency of specific MDR pathogens causing HAP and VAP may vary by hospital, patient population, type of ICU patient, and change over time.

### What are the Risk Factors for MDR Pathogens in VAP in ICU?

The incidence of VAP caused by MDR organisms has increased in the last decade and has been associated with increased cost of care, morbidity, and mortality. Data from the early 1980s show that about 50% of mechanically ventilated patient develop VAP within first 4 days after intubation and were due to non-MDR pathogens. However, several recent studies show no significant difference between causative organisms in both early and late VAP.^100^ Various factors like advanced age (> 60 years) and prior use of antibiotics have been consistently associated with increased risk of MDR organisms.^101,102^ In a prospective study done by Trouillet et al. in 135 cases of VAP, the three variables identified as risk factors for MDR VAP were the duration of mechanical ventilation (7 days or more) and prior use of broad-spectrum antibiotics (third-generation cephalosporins, fluoroquinolones, or imipenem).^103^ Renal replacement therapy and septic shock at admission were also found to be risk factors for MDR VAP.^104^ Higher Acute Physiology And Chronic Health Evaluation II (APACHE II) score on admission, pleural effusion, prior antibiotic treatment, illicit drug use, and tobacco are also found to be risk factors for MDR VAP due to MRSA.^105,106^ Similarly, vasopressor use, trauma, and neurological emergency were identified as additional risk factors for MDR VAP.^101^ Two studies show that systemic corticosteroid therapy has also been implicated as a risk factor for MDR VAP. However, both these studies do not mention the dose and duration for which corticosteroid therapy was used.^101,107^

#### Evidence Statement

The risk factors for VAP due to MDR organisms include age >60 years, duration of mechanical ventilation ≥7 days, prior antibiotic use within 3 months, the presence of severe sepsis or septic shock at the time of VAP, ARDS preceding VAP, renal replacement therapy prior to VAP and systemic corticosteroid therapy.

### What should be the Initial Combination of Empiric Antibiotic Therapy for VAP in ICU?

Inadequate or inappropriate therapy for VAP has been associated with higher mortality rates.^108^ A Cochrane review included four studies that compared monotherapy to combination antibiotic therapies for VAP. This analysis found no significant difference in the primary endpoint of all-cause mortality and clinical cure rate in intention-to-treat population and clinically evaluable population between monotherapy and combination therapy. Similarly, comparison of combination therapy with optional adjunctive antibiotics (amikacin, vancomycin, linezolid, aztreonam, ceftazidime, and tobramycin) did not find any difference in all-cause mortality, clinical cure rate in intention-to-treat population and clinical cure rate in the clinically evaluable population. No difference in all-cause mortality or clinical cure rate in intention to treat population was found when carbapenems were compared with non-carbapenems; however, carbapenems had a higher chance of clinical cure rate in the clinically evaluable population. This meta-analysis supports the use of a single antibiotic regimen with the understanding that resistance patterns may vary depending upon the local factors.^109^A similar meta-analysis by Infectious Disease Society of America (IDSA) also found no difference between combination therapy versus monotherapy, cephalosporins versus non-cephalosporin regimen, antipseudomonal penicillin versus non-antipseudomonal penicillin regimen and carbapenems versus non-carbapenem regimen. Among aminoglycoside versus non-aminoglycoside regimen, use of aminoglycoside regimen was associated with less chance of clinical response compared to the non-aminoglycoside regimen. When comparing quinolones versus non-quinolone regimen, adverse event rates were less with quinolone regimen [Risk Ratio 0.88 (0.78–0.99) with 95% CI].^85^A meta-analysis by Walkey et al.^110^ found that linezolid was not superior to glycopeptide antibiotics for the endpoints of clinical success, microbiological success, and mortality for patients with MRSA nosocomial pneumonia, without any significant difference in adverse events. However, another meta-analysis found more frequent gastrointestinal adverse effects with the use of linezolid.^111^

#### Evidence Statement

Use of antibiotic monotherapy and combination therapy for VAP have similar outcomes in patients who are not at risk for MDR pathogens. Commonly used antimicrobial agents include piperacillin-tazobactam, cefepime, levofloxacin, imipenem, and meropenem. Among antimicrobial agents, carbapenems have a higher chance of clinical cure than non-carbapenems. For treatment of VAP due to MRSA, glycopeptides and linezolid have similar clinical success; however, linezolid may be associated with a higher chance of thrombocytopenia and gastrointestinal adverse events.

#### Recommendations

- Among patients with VAP who are not at high risk of MDR pathogens and are in ICUs with a low prevalence of MRSA (<15%) and resistant gram-negative organisms (<10%), single antibiotic active against both MSSA and *Pseudomonas* is preferred over combination antibiotic (1A).
- Among patients with VAP who are at high risk of MDR pathogens or are in ICU with a high prevalence of MRSA (>15%) and resistant gram-negative organisms (>10%), an agent active against MRSA and at least two agents active against gram-negative organisms including *P. aeruginosa* is recommended (3A).
- Among patients with VAP who are not at high risk of MDR pathogens and are in ICU with a high prevalence of resistant gram-negative organisms (>15%) but low prevalence of MRSA (<10%), two agents active against gram-negative organism including *P. aeruginosa* is recommended (3A).
- Colistin is not recommended for routine use as an empirical agent in VAP. However, it may be used up front in the ICUs if there is a high prevalence of carbapenem-resistant *Enterobacteriaceae* (>20%) (UPP).
- In our country or areas with high endemicity of *tuberculosis*, use of linezolid may be restricted unless no suitable alternative is available (UPP).
- Fluoroquinolones and aminoglycoside should be cautiously used as monotherapy in VAP in our country as well as in other areas with high endemicity of *tuberculosis* (UPP).
- In ICU where the distribution of pathogen and antibiotic resistance pattern is known, empiric treatment should be designed accordingly, based upon patient risk factors for MDR pathogens (UPP).

### When to Give Antipseudomonal Drugs for VAP in ICU?

Antipseudomonal drugs are often started empirically in VAP when the risk factors for *Pseudomonas* infection are high. In a prospective surveillance study, it was found that the odds of developing *P. aeruginosa* VAP were 8 times higher in patients with prior *P. seudomonas* colonization than uncolonized patients.^112^ In a multicentre study, the independent risk factors for the presence of *P. aeruginosa* were the duration of hospital stay ≥ 48 hours before ICU admission, prolonged duration of ICU stay before enrollment > 9 days (highest quartile) versus ICU stay ≤ 4.8 days(lowest quartile).^113^ Risk factors of MDR *P. aeruginosa* include COPD, patients on mechanical ventilation > 8 days or patients with > 3 previous hospitalizations, and previous use of antibiotics.^114,115^

#### Evidence Statement

Prior use of antibiotics (most consistent association), prolonged duration of mechanical ventilation, and chronic obstructive pulmonary disease (COPD) have been identified as risk factors for MDR *P. aeruginosa* infection.

#### Recommendations

- Empiric treatment should be given to cover *Pseudomonas* if there are risk factors for MDR *Pseudomonas* infection (2A).
- In ICUs where gram-negative isolate resistance rate is low(<10% gram-negative isolate resistant to the agent being considered for monotherapy) and patients have no risk factors for antimicrobial resistance, one anti-pseudomonal antibiotic may be given (3A).
- In ICUs where gram-negative isolate resistance rate is high (>10% gram-negative isolate resistant to the agent being considered for monotherapy or not known), two antipseudomonal antibiotics from a different class to be given (3A).

### What should be the Duration of Antibiotic Treatment for HAP/VAP?

Prompt initiation of appropriate antimicrobial therapy is the mainstay of treatment of VAP. Selection of correct antimicrobial agent must be paired with an appropriate duration of therapy in order to optimally treat VAP/HAP. Several studies have evaluated the role of short duration antibiotic treatment in VAP/HAP. A study comparing 8 days therapy to 15 days therapy found no difference in mortality, relapses, mechanical ventilator-free days, organ failure free days and length of ICU stay while short course regimen was associated with more antibiotic-free days. However, gram-negative bacilli (*P. aeruginosa*) with short course regimen were more likely to have a relapse (40.6% *vs*. 25.4%).^116^ A randomized comparison of antibiotic discontinuation policy (discontinuation group) with treating physician teams policy (conventional group) found lower antibiotic duration in discontinuation group without any difference in a secondary episode of VAP, hospital mortality or ICU length of stay.^117^

#### Evidence Statement1

Short-course regimens for VAP are associated with significantly more antibiotic-free days without any significant difference in the duration of ICU or hospital stay, recurrence of VAP and mortality. Short-course regimens are associated with more recurrences in VAP due to non-fermenting gram-negative bacilli (NF-GNB).

#### Recommendations

- Short course (7–8 days) of antibiotic therapy should be used, in the case of VAP with good clinical response to therapy (1A).
- Longer duration (14 days) of antibiotic therapy should be considered, in case of VAP caused by NF-GNBs or is associated with severe immunodeficiency, structural lung disease (COPD, bronchiectasis, and interstitial lung disease), empyema, lung abscess, necrotizing pneumonia, and inappropriate initial antimicrobial therapy (3A).

### When should Anaerobic Cover be Added for VAP and Which is the Preferred Antimicrobial Agent?

Studies have reported the variable incidence of anaerobic organism isolation in nosocomial pneumonia occurring in mechanically ventilated patients as isolation of anaerobic bacteria requires adequate transport conditions and special growth media. In a retrospective study in 415 patients, factors associated with anaerobic infection were found to be altered level of consciousness and higher simplified acute physiology score (SAPS).^119^ Out of 163 isolates from VAP patients, only one was anaerobic (*Veillonella*) in a study done by PE Marik et al.^120^ Robert et al. evaluated the lower respiratory tract colonization by anaerobic bacteria in ICU patients on prolonged mechanical ventilation. Out of 26 patients, 22 were colonized by at least one bacterial strain and 5 patients developed VAP following colonization, and two were attributable to anaerobic bacteria.^121^

#### Evidence Statement

The incidence of anaerobic bacteria as the causative agent of VAP is 2–7%. Risk factors for VAP due to anaerobes are altered consciousness, aspiration pneumonitis and high simplified acute physiology score (SAPS).

#### Recommendations

- Empirical antibiotic regimen for VAP should not include coverage for anaerobic organisms routinely (2A).
- In the presence of risk factors for VAP due to anaerobic pathogens, anaerobic antimicrobial coverage should be added in an empirical regimen (2B).
- In patients with risk factors for anaerobic organisms, clindamycin or metronidazole should be added to empirical antibiotics regimen for VAP, if it does not include carbapenems (meropenem or imipenem) or piperacillin-tazobactam in the ongoing empirical regimen (UPP).

### When to Give Atypical Cover for VAP and Which is the Preferred Agent?

Atypical bacteria have been implicated as etiologic agents for VAP; however, no sufficient literature exists to assess the size of their role as a causative agent in VAP. The incidence of atypical bacteria is variable in various studies. A prospective study utilizing polymerase chain reaction (PCR) amplification method found 9 (15%) cases caused by atypical organisms (5 *Mycoplasma*, 3 *Legionella*, and 1 *Chlamydia*).^122^ Another study reported 6 cases of VAP due to *Legionella* among 26 patients with definite VAP.^123^ *M. pneumoniae* in 3 patients and *C. pneumoniae* in 2 patients were diagnosed among 100 VAP cases in a study by Apfalter et al.^124^ The risk factors for *Legionella* infection include the use of cytotoxic therapy and corticosteroids.^125^ If *L. pneumophila* is suspected organism for VAP, the combination antibiotic regimen should include a macrolide or a fluoroquinolone rather than an aminoglycoside.^126^

#### Evidence Statement

The incidence of atypical bacteria as causative agents of VAP is low (5–7.5%). Risk factors for VAP due to *Legionella* are *Legionella* colonization in hospital water supply, prolonged use of corticosteroids, cytotoxic chemotherapy, elderly, chronic renal failure, previous antibiotic use, granulocytopenia, and poor Glasgow coma score.

#### Recommendations

- Empirical antibiotic regimen for VAP should not include coverage for atypical organisms routinely (2A).
- In the presence of risk factors for VAP due to atypical bacterial pathogens, atypical antimicrobial coverage should be added to the empirical regimen (2B).
- The preferred atypical coverage in combination antibiotics regimen is fluoroquinolones (levofloxacin or moxifloxacin) or macrolides (azithromycin or clarithromycin) (UPP).

### Can Serum Procalcitonin be Used for De-escalation of Antibiotic Therapy in VAP?

Procalcitonin (PCT) is a polypeptide precursor to hormone calcitonin and is up-regulated from its normal low serum concentration in response to bacterial endotoxin or mediator of bacterial infection.^127^ Measurement of serum PCT has been investigated as a biomarker for the presence and persistence of infection, to guide decisions for initiation, de-escalation, and termination of antibiotic treatment. Delayed initiation of antibiotics in patients with sepsis contribute to increased mortality, while inappropriately prolonged use of antibiotics increases the risk of adverse events, including *Clostridium difficile* infection, and the development of antibiotic resistance. Various studies have evaluated the role of serum PCT in de-escalation of antibiotics. In a multicentric non-blinded RCT comparing guideline based antibiotic discontinuation with procalcitonin based antibiotic discontinuation, procalcitonin group had higher antibiotic-free days and reduction in the overall duration of antibiotic therapy through the ventilator-free days alive, ICU free days alive, length of hospital stay and mortality on 28 days were similar.^128^ PRORATA trial found that PCT-guided strategy to treat suspected bacterial infection in ICU could reduce antibiotic exposure by 2.7 days with no apparent adverse outcome.^129^ Two meta-analyses have also demonstrated increased antibiotic-free days in PCT-based strategies without negatively affecting the outcome.^130,131^ International guidelines differ on using procalcitonin for antibiotic de-escalation in VAP. American Thoracic Society guidelines suggest using PCT plus clinical criteria to guide the discontinuation of antibiotic therapy rather than clinical criteria alone.^85^ In contrast, European Respiratory Society (ERS) guidelines do not recommend the routine measurement of serial serum PCT levels to reduce the duration of antibiotic course in patients with HAP or VAP when the anticipated duration is 7–8 days although panel mention that they believe in measurement of serial serum PCT levels together with clinical assessment in specific clinical circumstances (such as severely immunocompromised patients, drug-resistant pathogens-NF-GNB, and initial inappropriate therapy).^132^

#### Evidence Statement

Use of procalcitonin to guide de-escalation of antibiotic treatment in patients with VAP is effective in reducing antibiotic exposure, without an increase in the risk of mortality or treatment failure.

#### Recommendations

- Serum procalcitonin may be used to guide the de-escalation of antibiotics in VAP when the anticipated duration of therapy is >7–8 days (1B).
- Serum procalcitonin levels (together with clinical response) should be used for de-escalation of antibiotic therapy in VAP in specific clinical conditions (severely immunocompromised patients, drug-resistant pathogens-NF-GNB, initial inappropriate therapy) (3A).

### How to Approach a Patient of Non-responding VAP?

Non-responding VAP or treatment failure in VAP is defined as the lack of improvement in clinical parameters (48–72 hours) with or without persistence of the infecting microorganism from the appropriate sample.^133,134^ Various clinical parameters such as the white blood cell count, measures of oxygenation and core temperature have been used in studies to define the normal pattern of resolution of HAP. In a prospective cohort study assessing the resolution of VAP, it was found that temperature normalizes within a median of 3 days and the ratio of arterial oxygen partial pressure to fractional inspired oxygen (PaO_2_/FiO_2_ ratio) improves by 2 days. ^135^ Another study evaluated the bacteriological and clinical efficacy of microbiological treatment of VAP among 76 VAP cases and demonstrated that appropriate antimicrobial therapy for VAP results in the control of the initial infection in 88% of the patients after day 3 of treatment.^136^ There are many implicated causes for non-resolution of VAP. These include wrong diagnosis (such as collapse, mass or pleural effusion), inappropriate initial treatment, delayed initiation of treatment, superinfection, the concomitant focus of infection or associated complications in the form of lung abscess, empyema or drug fever.^137,138^

#### Evidence Statement

Re-evaluation at 48–72 hours after the initial diagnosis of VAP is the most suitable time. By then the results of the initial microbial investigation are usually available, and treatment modification can be done. Evaluation of treatment response for VAP should be on the basis of clinical, laboratory, radiograph and microbiological results. Factors associated with treatment failure in VAP includes host factors (advanced age, immunosuppressed, chronic lung disease, ventilator dependence), bacterial factors (drug-resistant pathogens, opportunistic pathogens), therapeutic factors (inappropriate antibiotics, delayed initiation of therapy, insufficient duration of therapy, suboptimal dosing, inadequate local concentration of drugs), complications of initial VAP episode (lung abscess, empyema), other non-pulmonary infections or non-infectious mimics of pneumonia.

#### Recommendations

- Non-responding VAP should be evaluated for non-infectious mimics of pneumonia, unsuspected or drug-resistant pathogens, extrapulmonary sites of infection, and complications of pneumonia or its therapy and diagnostic testing should be directed to whichever of these causes is likely (2A).

### Catheter-related Bloodstream Infections (CRBSI)

Intravascular catheters are integral in the management of critically ill patients, especially those who require long-term medical care. They are most commonly used to access the vascular system for the delivery of medication, parenteral nutrition, a collection of blood samples and hemodynamic monitoring.^139^ CRBSI is defined as the presence of bacteremia originating from an intravenous catheter is a common complication leading to morbidity, mortality and adds to the cost of ICU stay. It is also the most common cause of nosocomial bacteremia in ICUs.^140^

#### Definition and Diagnosis

Catheter-related Bloodstream Infections (CRBSI) is defined as bacteremia or fungemia in a patient who has an intravascular device and one positive blood culture result obtained from the peripheral vein, clinical manifestations of infection (e.g., fever, chills, and/or hypotension), and no apparent source for bloodstream infection (other than the catheter). One of the following should be present, i.e., a positive result of semi-quantitative [>15 colony forming units (CFU) per catheter segment] or quantitative (>10^2^ CFUs per catheter segment) catheter culture, whereby the same organism is isolated from a catheter segment and a peripheral blood culture; simultaneous quantitative cultures of blood with a ratio 13:1 of CFU per milliliter of blood (catheter *vs*. peripheral blood); differential time to positivity (growth in a culture of blood obtained through a catheter hub is detected by an automated blood culture system at least 2 hours earlier than a culture of simultaneously drawn peripheral blood of equal volume).^141^ Catheter tip colonization (CC) is defined as significant growth of a microorganism (>15 colony-forming units) from the catheter tip culture.^141^ CRBSI rates are expressed as CRBSI rate per 1000 central line days. However, the suspicion of CRBSI arises in a patient using any intravascular catheter especially central venous catheter (CVC) who develops new-onset fever or chills, unexplained hypotension without any other localizing signs of infection.^140^

### What is the Incidence of Catheter Colonization and CRBSI?

Based on United States (US) data from National Nosocomial Infections Surveillance (NNIS) from 1990 to 1994, the CRBSI incidence (per 1000 catheter days) was 4.3 for respiratory intensive care units (RICU), 4.6 for medical-surgical ICUs, 7.3 for trauma ICUs and 12.2 for burn units.^142^ Data from NNIS from January 1992 through June 2004 showed that the median rate of CRBSI in ICUs of all types ranged from 1.8 to 5.2 per 1000 catheter days,^143^ whereas another survey in 2010 showed the mean incidence up to 1.76 per 1000 catheter days, suggesting a decreasing trend.^144^

Data from Extended Prevalence of Infection in Intensive Care Study (EPIC 2) showed an overall point prevalence of 4.7 per 1000 catheter days.^145^ A prospective observational study by Lorente et al. showed the incidence of CC as 6.04% and of CRBSI to be 2.79 per 1000 catheter days.^146^ Other studies have shown the global incidence of CC to be 1.4–20 % while that of CRBSI to be 2.4–12.5 per 1000 catheter days.^147-150^ Majority of these studies have shown CVCs as the commonest cause for CRBSIs. The data from India suggest a higher incidence of CC and CRBSI. In a study by Mittal et al. CC was found in 59% catheters with CRBSI rate of 9.5 per 1000 days.^139^ Others have shown the incidence of CC as 18–42% while of CRBSI is 1 to 16.1 per 1000 catheter days.^151,152^

#### Evidence Statement

The global incidence of CC ranges from 1.4 to 19.4% whereas CRBSI incidence ranges from 2.4 to 12.5%. The incidence of CC is higher in Indian ICUs ranging from 18% to as high as 59%, whereas the incidence of CRBSI is up to 16.1 per 1000 catheter days.

### What are the Risk Factors for CRBSI?

The incidence of CRBSI varies considerably according to various factors such as the type of catheter (single or multi-lumen), duration of indwelling catheters, the frequency of catheter manipulation, and patient-related factors such as age, underlying disease, and severity of illness. In a retrospective study in 73 events of CRBSI, major risk factors found were advanced age, long-term indwelling catheter, parenteral nutrition, diabetes mellitus (DM), and APACHE II score >23, and more than three underlying diseases. Multivariate analysis showed that an APACHE II score >20 and more than three underlying diseases were independent factors associated with CRBSI occurring within 14 days of CVC insertion.^153^ Duration of the catheter is an important parameter and catheter duration >14 days is an independent risk factor for CRBSI.^147,150,154-157^ The risk for CRBSI is higher when the interval time for dressing change is longer than 48 hours irrespective of the dressing material (permeable or semi-permeable).^149^ Use of transparent dressings, regular change of dressings, total parenteral nutrition, and use of three-way cannulas have not been consistently associated with increased risk for CRBSIs.^149,154^ Regarding hemodialysis (HD) catheters, prospective data by Caylan et al. in 248 patients with HD catheters have shown acute renal disease, administration of antibiotics at the time of catheterization, insertion in the femoral vein, emergency situation for catheter insertion, high number of catheter manipulation, and inadequate hand hygiene prior to catheter manipulations as risk factors of CRBSI.^158^ Catheter-related candidemia should be suspected in patients with any of the following risk factors: total parenteral nutrition, prolonged use of broad-spectrum antibiotics, hematologic malignancies, and receipt of bone marrow or solid-organ transplant, femoral catheterization, or colonization due to *Candida* species at multiple sites.^141^

#### Evidence Statement

Longer indwelling catheter duration, immunosuppression, diabetes mellitus, sepsis at the time of insertion, multi-lumen catheters and APACHE >23 are important risk factors for CRBSI. APACHE at admission, renal failure, central venous catheterization, and steroid therapy are important risk factors for fungal CRBSI.

### What are the Common Organisms Causing CRBSI and their Antibiotic Susceptibility?

Apart from the severity of the patient's clinical disease and risk factors for infection, the initial choice of antibiotics will also depend on the likely pathogens and their susceptibility patterns. According to the available literature, certain organisms should always be considered, apart from taking local epidemiology into account. National Nosocomial Infections Surveillance (NNIS) survey of nosocomial infections from 1990 to 1999 showed coagulase-negative *Staphylococcus* (CONS), *Staphylococcus aureus* and *Enterococcus* as common organisms while *Candida* albicans accounted for 5% of the CRBSI. A large proportion of CONS isolates were methicillin resistant, and the incidence of MRSA and vancomycin-resistant *Enterococcus* (VRE) was 54.5% and 25.9% respectively.^142^ According to NNIS 2004 data, 87% of CRBSI were monomicrobial, out of which 65% were gram-positive organisms, 25 % were gram-negative organisms and 9.5% were fungi, with CONS, *Staphylococcus*, and *Candida* being the common organisms.^159^ During this period, there was 12% increase in VRE and 11% increase in MRSA. There was a marked increase in ESBL producing *Klebsiella* with 47% increase in overall incidence. The proportion of CRBSI due to gram-negative organisms like *Pseudomonas, Acinetobacter* and *Klebsiella* is also on rising trends according to recent studies. In a recent observational study, CRBSIs due to *Pseudomonas* and *Acinetobacter* were 22.2% and 20% respectively.^147^ This rise in gram-negative organisms has been found in various studies from India as well.^152,153,160,161^ In Indian ICUs the MRSA incidence ranges from 30 to 87%, and that of VRE is as high as 25%.^160,161^ Incidence of ESBL producing organisms has also increased with some studies showing all isolates to be ESBL producing.^162^ The proportion of CRBSI caused by fungi varies among different studies and usually ranges from 4.4% to 20% and mostly was due to *Candida albicans*.^161,163^ However, a prospective observational study from 27 Indian ICUs found *Candida tropicalis* (41.6%) as the most common cause of fungemia followed by *Candida albicans* (20.9%) and *Candida parapsilosis* (10.9 %). Majority of *C. tropicalis* isolates were sensitive to amphotericin B (99.0%), azoles (90.1%), fluconazole (97.4%) and echinocandins (94.2 %).^164^

#### Evidence Statement

Coagulase-negative staphylococci (CONS), *S. aureus, Enterococcus*, and *Candida* species are the common organisms accounting for the majority of the CRBSIs. A large proportion of *Staphylococcus aureus* and CONS are methicillin resistant ranging from 11 to 87 %. There is an increased incidence of CRBSI due to gram-negative organisms (most of which are ESBL producers) and *Candida* especially the non-*albicans Candida*.

### What is/are the Empiric Antibiotic(s) of Choice for CRBSI in ICU?

Empiric treatment, when indicated, should provide coverage against the most frequent organisms causing CRBSI, i.e. gram-positive as well as gram-negative organisms. Vancomycin, teicoplanin, and linezolid are considered the initial drugs of choice for empiric treatment for gram-positive organisms as the incidence of methicillin resistance is high among CONS and *S. aureus*. A recent meta-analysis by Li et al. included 7 RCTs comparing linezolid with vancomycin in 5376 patients with MRSA.^165^ The clinical cure rate in linezolid group was higher than that of vancomycin group after treatment (OR 1.85; 95% CI: 1.33–2.59, p < 0.001) and at follow-up (OR 1.49; 95% CI: 1.17–1.91, p = 0.001). However, linezolid monotherapy has not been recommended for empirical treatment of patients with suspected CRBSI.^166^ Teicoplanin is a safe and effective alternative to vancomycin considering the lesser toxicity and once daily dosing.^167^ Quinupristin-dalfopristin and daptomycin may be alternative drugs effective in MRSA bacteremia and enterococci showing comparable results with vancomycin in RCTs.^168,169^ Dalbavancin is another drug belonging to same class as vancomycin and when used in weekly doses, has shown higher success rate than vancomycin for treatment of CRBSI.^170^ For treatment of VRE, a significantly lower mortality rate and trend towards better clinicomicrobiologic response has been seen using linezolid as compared to quinupristin-dalfopristin.^171^ Apart from gram positive coverage, an antimicrobial agent with activity against aerobic gram-negative bacilli should be added to the empiric coverage of CRBSI. The appropriate options include aminoglycosides, aztreonam, third-generation cephalosporins with antipseudomonal activity, fourth-generation cephalosporins, piperacillin-tazobactam or quinolones.^141^ In patients with risk factors for candidemia empiric treatment against *Candida* is sometimes considered. Caspofungin and fluconazole have equal cure rates in culture positive *Candida* infections with no difference in mortality as compared to amphotericin B.^172,173^

#### Evidence Statement

Vancomycin, teicoplanin, linezolid, and daptomycin are effective in the treatment of CRBSI due to MRSA and MR-CONS. Fourth-generation cephalosporin, carbapenem or beta-lactam/beta-lactamase combination like piperacillin/tazobactam and aminoglycosides might be used for gram-negative organisms causing CRBSI. Caspofungin and fluconazole are equally effective as amphotericin-B for treatment of candidemia.

#### Recommendations

- Empirical antibiotic regimen for CRBSI should include coverage for both gram-positive and gram-negative organisms (2A).
- Vancomycin or teicoplanin is the recommended first-line drug for the empiric treatment of CRBSI for MRSA and MR-CONS while linezolid and daptomycin are good alternative agents (2A).
- Empiric coverage for gram-negative bacilli should include a fourth-generation cephalosporin, a carbapenem, or a β-lactam/β-lactamase inhibitor combination, with or without an aminoglycoside. (UPP)
- An echinocandin or fluconazole should be used as empirical antifungal agents for the treatment of suspected central line-associated candidemia (2A).

### What should be the Duration of Antibiotic Treatment for CRBSI?

Optimum duration of antibiotic treatment to the bare minimum required to treat infections is a reasonable approach to reduce the prevalence of resistance to antibiotics. No significant differences in clinical cure, microbiologic cure and survival were detected among bacteremic patients receiving shorter (5–7 days) versus longer duration (7–21 days) of antibiotic therapy in a meta-analysis.^174^ There was 5–10% relapse rate after short-course therapy for *Staphylococcus aureus* catheter-associated bacteremia suggesting that short-course therapy is acceptable for uncomplicated infections. In case of complicated *S. aureus* infections like infective endocarditis, longer duration (4–6 weeks) of treatment is required. Studies have shown similar response irrespective of duration of therapy in gram-negative infections as well. Regarding the duration of empirical antifungals for CRBSIs, there has been no comparative studies but based on the consensus, approximately 14 days of empirical antifungals are recommended.

#### Evidence Statement

Short duration (<14 days) of antibiotics is as effective as longer duration (>14 days) for uncomplicated *Staphylococcus aureus* bacteremia. Complicated bacteremia due to *S. aureus* or those associated with endocarditis should receive longer duration. For gram-negative bacteremia, seven days of antibiotics are sufficient. In responding patient with uncomplicated CONS infection, 5–7 days therapy is considered optimum. Minimum 14 days treatment with antifungals is required for fungal CRBSI.

#### Recommendations

- Minimum 2 weeks antibiotics should be given for uncomplicated and 4–6 weeks for complicated *Staphylococcus aureus* CRBSI and infective endocarditis (2A).
- Minimum 7 days of antibiotics should be given for gram-negative CRBSI (2A).
- Five to seven days antibiotics are recommended for CONS bacteremia (3A).
- For suspected fungal CRBSI, antifungal therapy for at least 14 days is recommended (UPP).

### Empirical Antibiotics for Urinary and Urogenital Sepsis in ICU

Urogenital infections in patients in the ICU include urinary tract infection (UTI) and prostatitis in males. The clinical spectrum of UTI includes asymptomatic bacteriuria and funguria to pyelonephritis, and urosepsis with or without obstructive uropathy. Urinary tract infections are the fourth most common type of healthcare-associated infection.^175^ UTI additionally account for more than 12% of infections reported by acute care hospitals. About 12–16% of hospitalized adults have indwelling urinary catheter at some time during their hospitalization. Each day the indwelling urinary catheter is in place, there is a 3–7% increased risk of acquiring a catheter-associated urinary tract infection.^176^ UTIs in ICU have different microbiology and higher resistance rates than UTI occurring outside ICU. Urinary tract infection is defined as significant bacteriuria in a patient with symptoms or to the urinary no alternate source. Significant bacteriuria in a patient without symptoms or signs attributable to the urinary tract is defined as asymptomatic bacteriuria.

Catheter-associated urinary tract infection (CA-UTI) is defined as an infection occurring in a person whose urinary tract is currently catheterized or has been catheterized within the previous 48 hours with urethral, suprapubic or intermittent catheterization. It is characterized by symptoms and signs suggestive of UTI with no other obvious source and a urine sample (from the urinary catheter, or midstream urine for catheter duration less than 48 hours) demonstrating more than 1000 CFU per mL. On the other hand, catheter-associated asymptomatic bacteriuria refers to patients with urethral, suprapubic or intermittent catheterization with urine culture positivity (>10^5^ CFU/mL) without any signs or symptoms attributable to UTI. According to CDC, CA-UTI is defined as a UTI in patients with an indwelling urinary catheter that had been in place for >2 days on the date of event (day of device placement = D1) and was either present for any portion of the calendar day on the date of event or removed the day before the date of event. The patient should have at least one of the following signs or symptoms: fever, suprapubic tenderness, costovertebral angle pain or tenderness, urinary urgency, urinary frequency, and dysuria along with urine culture with no more than two species of organisms identified at least one of which is a bacterium of ≥10^5^ CFU/mL.^177^

### What is the Incidence of UTI in ICU? What are the Common Organisms and Risk Factors for UTI in ICU?

The incidence of UTI ranges from 5 to 23 per 1000 catheter days as reported from various observational studies from the West.^178-183^ In an observational study, Tay et al. from Singapore reported the incidence of UTI from mixed ICU to be 13.7% in patients admitted for more than 48 hours, with the incidence of *Candida* being about 34%.^184^ The organisms causing UTI were *Klebsiella* (7%), *E. coli* (7%), polymicrobial (37%) and others (7%). Female gender, prior antibiotic exposure, duration of ICU and urinary catheter were identified as risk factors for UTI. In a prospective observational study from China, Xie et al.^185^ reported the incidence of UTI to be 25.5 per 1000 catheter days. Fungi (21.3%) were the most common cause of UTI followed by infection with *E. coli* (17.02%) and *Pseudomonas* (10.64%). The risk factors for CA-UTI were the duration of the catheter for > 7 days, benign prostatic hypertrophy and > 5 days antibiotic duration. *Pseudomonas* showed absolute resistance to ciprofloxacin, amikacin, ceftazidime, and meropenem. A prospective study by Leone et al. reported an incidence of UTI to be 9.6%. The common organisms isolated were *E. coli* (39%), *Pseudomonas* (22%) and *Enterobacter* (15%).^186^ Duration of catheterization, length of ICU stay, advanced age, female gender, and disease severity score were identified as risk factors for CA-UTI. Similar findings were reported by various studies from western world.^187–190^ In the ENVIN registry, gram-negative bacteria were responsible for more than half of the cases of UTI (56.7%) with *E. coli* being the commonest organism isolated (26.7%). Fungal infection was second most common (25.4%) with *Candida* albicans as most common fungus isolated.^191^ In a prospective study by Agarwal et al.^192^ from Northern India, the organisms causing UTI in ICU included *Acinetobacter* (34.8%), *Pseudomonas* (23.8%) and *E. coli* (15.2 %). Length of ICU stay, renal failure and total parenteral nutrition (TPN) were reported as risk factors for UTI. In a prospective observational study by Habibi et al.^193^ including patients with greater than 48 hours of ICU stay, most common causes of UTI were *Candida* spp. (90%) followed by *Pseudomonas* (14%) and *E. coli* (10%). Increased ICU stay and catheterization were identified as risk factors for UTI. Gupta et al.^194^ reported the incidence of UTI in patients admitted in ICU to be 28%. *E. coli* was the most common organism responsible for UTI (30.8%). Longer ICU stay, catheterization and prior antibiotics use were identified as risk factors for UTI. In a retrospective review by Sahu et al.,^195^ incidences of UTI reported was 6.9%. Identified risk factors included longer ICU stay and catheterization.

#### Evidence Statement

The incidence of CA-UTI ranges from 5% to 30% of all ICU admissions. The most common organism causing UTI in ICU are gram-negative bacteria (*E. coli, Klebsiella*) and fungi (especially *Candida*). Risk factors for UTI in ICU include the duration of catheterization, length of ICU stay, prior antibiotic use, higher disease severity score, and female gender.

### What is the Empirical Antimicrobial Agent of Choice for Treating UTI in ICU?

A systematic review and meta-analysis by Vardakas et al.^196^ included 21 studies and 1584 patients with ESBL producing *Enterobacteriaceae* bacteremia. He compared the mortality associated with carbapenems and alternative antibiotics (beta-lactams/beta-lactamase inhibitors) for the treatment of patients with ESBL-positive *Enterobacteriaceae* bacteremia. No statistically significant differences in mortality were found between carbapenems and beta-lactams/beta-lactamase inhibitors administered as a definitive or empirical treatment for UTI.

In an observational study on gram-negative UTI in hospitalized patients, all isolates were susceptible to carbapenems, with 70–80% susceptible to fluoroquinolones, aminoglycosides, and cefepime. Organisms were resistant to amoxicillin, amoxicillin-clavulanic acid and co-trimoxazole. Gram-negative Enterobacteriaceae was also resistant to the second and third generation cephalosporins.^197^ Another prospective study reported an increase in the frequency of gram-negative Enterobacteriaceae and *S. aureus* in catheter-associated nosocomial UTI over 10 years, with high sensitivities to amikacin, imipenem, and piperacillin-tazobactam (72.0%, 77.5%, and 76.1%, respectively). Lower susceptibility to third-generation cephalosporins and ciprofloxacin (55.2% and 45.0% respectively) were reported. Gram-positive organisms showed high susceptibility to teicoplanin and vancomycin (91.1% and 87.9% respectively) and low susceptibility to ampicillin and ciprofloxacin (24.1% and 25.5% respectively).^198^ Habibi et al.^193^ from northern India reported the antibiotics resistance pattern of gram-negative bacteria causing UTI. In this study, the bacteria were resistant to ceftazidime and netilmicin. Cefoperazone–sulbactam resistance was least common among gram-negative organisms. Sahu et al.^195^ reported the least resistance to tigecycline, colistin, and carbapenems among the gram-negative Enterobacteriaceae. One study reported antibiotic susceptibility pattern in gram-negative Enterobacteriaceae and most of the isolates were susceptible to carbapenems, amikacin and levofloxacin.^199^ In a RCT, three antibiotics piperacillin-tazobactam, cefepime and ertapenem were compared in terms of clinical and microbiological cure rate and 28 days mortality for treatment of ESBL producing *E. coli*. Both cure rates were high for piperacillin-tazobactam and ertapenem. Cefepime was found least effective in terms of both cure rate and prevention of mortality.^200^ In a prospective study, 89.2% of urinary culture isolates were sensitive to fosfomycin; 89.2% of gram-negative bacilli including Enterobacteriaceae were also susceptible.^201^ Patel et al.^202^ evaluated *in vitro* activity of fosfomycin against urinary tract Enterobacteriaceae; 79.16% of the isolates were susceptible to fosfomycin with 92% susceptibility in ESBL producing Enterobacteriaceae and 72.34% in carbapenem resistant Enterobacteriaceae (CRE). MDR Enterobacteriaceae with diverse resistance mechanisms, including ESBL and CRE were found to be susceptible to fosfomycin.^5^

#### Evidence Statement

There has been a trend towards increasing prevalence of extended-spectrum beta-lactamase producing gram-negative bacteria in the urinary cultures of catheter-associated UTI. Aminoglycosides, beta-lactams along with a beta-lactamase inhibitor as well as carbapenems and fosfomycin have good efficacy in catheter-associated UTI. The susceptibility for fluoroquinolones is decreasing over time among organisms isolated from nosocomial UTI. *Candida* species isolated from the patients with UTI show sensitivity to fluconazole.

#### Recommendations

- Initial choice of antibiotics should cover for ESBL producing gram-negative organisms and includes aminoglycosides, beta-lactam along with a beta-lactamase inhibitor or carbapenems (2A).
- In the initial empirical regimen for UTI, antibiotics against gram-positive organisms are not recommended (3A).
- In appropriate clinical settings, antifungals should be considered in the empirical regimen (3B).

### Acute Infective Diarrhea, Antibiotic-induced Diarrhea, and Clostridium difficile-associated Diarrhea in the ICU

Diarrhea is defined as the passage of more than three liquid stools in a day.^206^ Nosocomial diarrhea is defined as one which arises after 3 or more days of admission to the hospital.^207^ Up to 30% of patients in hospital develop nosocomial diarrhea and majority of which have non-infectious etiology. Among infectious causes, *Clostridium difficile-*associated diarrhea is the most common.^208^ Overall the incidence of diarrhea in intensive care unit varies between 15% and 40% in different studies where most cases have a non-infectious or multifactorial etiology.^209^

### Etiology of Diarrhea in the ICU

Non-infectious etiologies of diarrhea are commoner in ICU, including enteral feeding, stool impaction and drugs (laxative, prokinetics, histamine antagonists, potassium supplements).^210^ Other factors such as sepsis, antibiotic therapy, and hypoalbuminemia increase the likelihood of diarrhea.^211^ *Clostridium difficile* is the most common infectious agent associated with diarrhea in the ICU.^212^ Infectious etiology is suspected if diarrhea is associated with fever, leukocytosis, vomiting, severe abdominal pain, mucus or blood in the stool.^213^ Clinical presentation may range from mild infection to life-threatening illness with the pseudo-membrane formation, toxic megacolon, colonic perforation, sepsis or even death.^212^ The American College of Gastroenterology (ACG) have proposed a severity scoring system for *Clostridium difficile* infection.^214^

### Diagnosis of Acute Infective Diarrhea in the ICU

*Clostridium difficile* accounts for the majority of infectious diarrhea in the ICU. Most commonly employed screening test is enzyme immunoassay (for Toxin A and B).^215^ Gold standard for diagnosis remains cytotoxin neutralization assay (CCNA) and toxigenic culture, with the latter being more sensitive. ^215^ Other diagnostic tests include stool glutamate dehydrogenase and polymerase chain reaction techniques. As per *Clostridium difficile* infection (CDI) severity index, CDI is defined as severe and complicated if it is associated with any of the following, i.e., hypotension, fever(≥38.5° C), ileus or significant abdominal distension, mental status changes, leucocytosis (≥ 35,000 cells/mm^3^), leucopenia (<2,000 cells/mm^3^), lactic acidosis (>2.2 mmol/L) or end organ failure. Severe disease refers to CDI with hypoalbuminemia (< 3 g/dL) along with either abdominal tenderness or leucocytosis (WBC ≥ 15,000 cells/mm^3^). Mild to moderate disease refers to CDAD not satisfying above criteria. *Clostridium difficile* infection (CDI) is a leading cause of hospital-associated gastrointestinal illness and places a high burden on our health-care system. Patients with CDI typically have extended lengths-of-stay in hospitals, and CDI is a frequent cause of large hospital outbreaks of disease.^216^

### What are the Common Organisms Causing Acute Infective Diarrhea in the ICU?

In a large prospective study, it was reported that infectious etiologies accounted for 9.2% of cases of acute diarrhea in a mixed general intensive care unit.^217^ *Clostridium difficile* was the most common infective cause accounting for 97 out of the 112 patients in the above study.^217^ In Indian studies, the incidence of CDI was around 16 to 17%.^218,219^ Other organisms include *Pseudomonas* aeroginosa and *Staphylococcus* which have been associated with sporadic outbreaks of diarrhea in the intensive care unit.^220,221^ Viruses are another important cause of infective diarrhea in ICU. *Norovirus* was isolated in 5.7% cases in one study.^217^ Outbreaks of viral diarrhea due to *norovirus* have also been reported in ICU settings.^222^

#### Evidence Statement

The incidence of diarrhea in the ICU ranges from 12.9 to 38%. Majority of the cases of diarrhea in ICU are non-infectious in etiology. *Clostridium difficile* is responsible for the majority of infectious cases of diarrhea in ICU.

### What are the Empirical Antibiotics of Choice for Treating Acute Infective Diarrhea in the ICU?

There is a lack of studies that evaluate the use of empirical antibiotics in patients with diarrhea in the ICU setting. In a prospective study evaluating the utility of metronidazole in presumptive *Clostridium difficile* diarrhea involving 70 patients, 18 (25%) were subsequently proven to have *Clostridium difficile*-associated diarrhea (CDAD) whereas 49 (68%) patients had no identifiable cause. Patients who had CDAD had significant improvement in symptoms as compared to those without it.^223^ The American College of Gastroenterology guidelines assert that patients with diarrhea in the ICU who have a strong pretest suspicion of CDI should receive empirical treatment pending the results of laboratory testing, and even in patients with negative testing, as the negative predictive value of existing tests for CDI is insufficiently high to rule out the infection.^214^

#### Evidence Statement

Empirical use of metronidazole in patients with diarrhea suspected due to *Clostridium difficile* in ICU setting results in significant symptomatic improvement.

#### Recommendations

- We recommend that empiric metronidazole be used for therapy of patients with acute diarrhea in the ICU with suspected *Clostridium difficile* infection (3A).

### What are the Risk Factors for the Development of CDI or CDAD?

Various factors associated with increased risk of CDI include prior antibiotic use, advanced age, prolonged ICU or hospital stay, immunosuppression, proton pump inhibitor use, and enteral feeding. In a recent meta-analysis, previous antibiotic use of second-generation cephalosporins (OR 2.23, 95% CI 1.47–3.37), third-generation cephalosporins (OR 3.20, 95% CI 1.80–5.71), fourth-generation cephalosporins (OR 2.14, 95% CI 1.30–3.52), carbapenems (OR 1.84, 95% CI 1.26–2.68), clindamycin (OR 2.86, 95% CI 2.04–4.02), cotrimoxazole (OR 1.78, 95% CI 1.04–3.05), fluoroquinolones (OR 1.66, 95% CI 1.17–2.35) and penicillin combinations (OR 1.45, 95% CI 1.05–2.02) increased the risk of CDAD.^224–234^

Advanced age has been shown to be associated with increased incidence of CDI.^219,235-237^ Other risk factors for CDI/CDAD include longer ICU stay, enteral feeding, prolonged mechanical ventilation, and immunosuppression.^212–214,218,225,236-241^ Proton pump inhibitors (PPI) have been shown to be independent risk factor for CDAD, possibly due to elevated gastric pH accelerating conversion of *C. difficile* spores to vegetative forms.^219,242-245^

#### Evidence Statement

Risk factors for the development of CDI include prior antibiotic therapy, advanced age, prolonged ICU/hospital stay, immunosuppression, proton pump inhibitors, and enteral feeding. Cephalosporins, clindamycin, fluoroquinolones, carbapenems, and penicillin derivatives are the commonly implicated antibiotics for CDAD/CDI.

### What is the Recommended Treatment for CDI/CDAD: Which Antibiotics and for what Duration? Should Offending Antibiotics be Stopped? What is the Role of Probiotics in the Treatment of CDAD? How should Recurrent Clostridium difficile Infection be Treated?

While certain antibiotics have a propensity to cause CDI, antimicrobial therapy against *C. difficile* has been found to be successful in treating CDI in a clear majority of cases. In a Cochrane review that included 22 randomized controlled trials with 3215 participants, four RCTs directly compared vancomycin and metronidazole for the symptomatic cure of CDI.^246,247-250^ It was found that vancomycin was modestly superior to metronidazole for the treatment of CDI with a moderate quality of evidence. However, metronidazole has a much lower cost and an acceptable efficacy for this indication. Fidaxomicin (a newer oral antibiotic with minimal absorption) was non-inferior to vancomycin for treatment of CDI in a multicenter randomized trial.^251^ It was more effective than vancomycin in achieving clinical cure when patients were receiving concomitant antibiotics for concurrent infections.^252^ There are no direct comparisons between fidaxomicin and metronidazole, however, a network meta-analysis including studies that compared fidaxomicin with vancomycin and vancomycin with metronidazole concluded that fidaxomicin was superior to the other two agents for the sustained cure of CDI.^253^ Clinical cure rate following oral teicoplanin for management of CDI was comparable with oral vancomycin for management of CDI (96.2% *vs*. 100%, p = 0.56).^254^ Similar cure rates were reported on comparing teicoplanin with both metronidazole and vancomycin for management of CDI.^255^ A pertinent question is whether the offending antibiotic should be stopped during treatment of *C. difficile* infection. A retrospective review of 246 patients found that the use of implicated antibiotics after the completion of CDI treatment was significantly associated with recurrence of CDI compared to no antimicrobial use [odds ratio (OR) 3.02; 95% CI, 1.66–5.52]. On the contrary, the use of the implicated antibiotic during the CDI therapy was not associated with recurrent CDI (OR 0.79; 95% CI, 0.40–1.52).^256^ This suggests that treatment of the primary infection may continue, if necessary, with appropriate antibiotic under the cover of CDI therapy.

Use of probiotics in addition to antibiotics for treatment of CDI showed that probiotics reduced the rate of recurrence in patients with recurrent CDI but not in patients with an initial episode.^257^ In a systematic review use of probiotics in the treatment of CDI was not effective.^258^ Whilst probiotics are unsuccessful in the treatment of CDI, they have been found to be beneficial for preventing CDI in patients receiving antibiotics. In a review of 26 RCTs, probiotics (including *Lactobacillus, Saccharomyces*, and combinations) significantly reduced the risk of developing CDAD by 60.5% (RR = 0.395; 95% CI 0.294–0.531; p <0.001).^259^

Recurrent CDI occurs in up to one-third of the patients and is associated with considerable morbidity and costs. A systemic review that included three studies comparing vancomycin with metronidazole, reported that vancomycin and metronidazole are equally effective in the treatment of recurrent CDI.^260–263^ Addition of *Saccharomyces boulardii* to vancomycin significantly decreased the recurrence rate (16.7% *vs*. 50%, p = 0.05).^263^ Fidaxomicin was more effective as compared to vancomycin for recurrent CDI (RR 1.86, 95% CI 1.04–3.31, p = 0.04).^251,264^ Fecal microbiota transplantation has also been compared to drug therapy for treatment of recurrent CDI. It was found that vancomycin therapy with a duodenal infusion of donor feces had relapse free cure rate of 93.8% as compared to 30.8% and 23.1% in vancomycin with bowel lavage and vancomycin therapy alone respectively.^265^

#### Evidence Statement

Both metronidazole and oral vancomycin have similar efficacy in the clinical and bacteriologic cure of CDI. Use of implicated antibiotic after completing the treatment of CDI is associated with increased risk of recurrence of CDI. There is insufficient evidence to justify the use of probiotics as an adjunct to antibiotics in the treatment of CDAD. In a single RCT, fecal microbiota transplantation was found to be highly efficacious for treatment of recurrent CDI.

#### Recommendations

- We recommend metronidazole as the first line treatment of mild to moderate CDI/CDAD (1A).
- We recommend oral vancomycin as the first line treatment of microbiologically proven severe CDI/CDAD (1A).
- We recommend oral vancomycin as the treatment of recurrent CDI/CDAD infection (2A).
- We recommend fecal microbiota transplantation as an alternate treatment of recurrent CDI/CDAD infection (2A).
- We recommend that implicated antibiotics should be discontinued as soon as clinically feasible (2A).
- We recommend against the use of probiotics as an adjunct for the treatment of CDI/CDAD (2A).
- We recommend the addition of vancomycin to a patient with microbiologically proven CDI/CDAD if the patient is already on metronidazole or has no clinical response to metronidazole within 3–4 days. (UPP)

## ABDOMINAL INFECTIONS IN ICU

### Acute Pancreatitis and Infected Pancreatic Necrosis

Acute pancreatitis (AP) is the inflammatory condition of the pancreas characterized clinically by abdominal pain and raised serum levels of pancreatic enzymes.^266^ Majority of the cases are caused by cholelithiasis and chronic alcohol consumption.^267,268^ Depending on the severity, AP is divided into mild, moderate and severe. Severity of pancreatitis is based upon the presence of organ failure and complications of acute pancreatitis either local or systemic.^269^ Local complications include peripancreatic fluid collections and pancreatic or peripancreatic necrosis (sterile or infected) whereas systemic complications include failure of an organ system (respiratory, cardiovascular, or renal) and exacerbation of a preexisting disorder (e.g., chronic obstructive pulmonary disease, heart failure, or chronic liver disease).^270^ Patients with mild AP have no evidence of organ failure, local or systemic complications. Moderately severe AP is defined by the presence of transient organ failure lasting less than 48 hours with or without local and systemic complications. Persistent organ failure for more than 48 hours associated with local and systemic complications defines severe AP (SAP).^269,271^ About 20 –30% of patients with AP develop acute necrotizing pancreatitis.^272,273^ Pancreatic necrotic tissue may remain sterile (~70%) or may get infected (~30%). The severity of necrotizing pancreatitis is determined on the basis of the extent of parenchymal involvement by necrosis (i.e.,<30%, 30–50%, and >50%).^274^ Infected pancreatic necrosis is associated with higher mortality as compared to sterile necrosis.^275,276^ Thus, early recognition and the institution of appropriate therapy is necessary. Treatment options include administration of antibiotics and surgical intervention if there is no response to antibiotics.^277,278^

### What are the Incidence, Risk Factors, and Microbiology of Pancreatic Infection Following Acute Pancreatitis?

#### Incidence and Risk Factors for Infected Pancreatic Necrosis

The incidence of infected pancreatic necrosis (IPN) in patients with acute pancreatitis varies from 12% to 37% depending upon the patients included (AP *vs*. SAP) and diagnostic modality used for IPN.^279-282^ Patients with necrotizing pancreatitis are more prone to develop a pancreatic infection and organ failure.^275,276^ Greater the extent of necrosis more likelihood of IPN. In a retrospective review of 300 patients of AP, pancreatic infection and organ failure were directly related to the extent of pancreatic necrosis.^281^ In a prospective single-center study that included 204 patients of AP, pancreatic necrosis of more than 50% was significantly associated with the development of pancreatic infection and multiorgan failure.^282^ In a prospective observational study from India, similar findings were reported.^279^ Patients of AP can develop organ failure either during the early phase (<1 week) known as primary organ failure or during a later phase of AP (>1 week) known as secondary organ failure.^283,284^ In a prospective observational study in 805 patients of acute pancreatitis, the presence of primary organ failure was associated with mortality of 15.8% and was a risk factor for the development of infected pancreatic necrosis in 76% of patients.^285^

#### Evidence Statement

The incidence of pancreatic infection following acute pancreatitis ranges from 12 to 37%. Presence of pancreatic necrosis of >50% is a major risk factor for pancreatic infection following acute pancreatitis. Primary organ failure predicts the development of infective pancreatic infection in patients with acute pancreatitis.

### Microbiology of Pancreatic Infection Following Acute Pancreatitis

Enteric gram-negative bacteria including *E. coli, Klebsiella, Pseudomonas*, and Enterobacteriaceae are the most common organisms isolated from IPN.^286,279,287^ It has been demonstrated that translocation of enteric bacteria (from the gut) is the main source of infection in necrotizing pancreatitis.^288,289^ A recent prospective observational study from India evaluated 209 patients with AP; 108 (52%) developed infected pancreatic necrosis (IPN). Polymicrobial infection was seen in 51% patients. Most common GNB isolated was *E. coli* (32%), *E. faecium* was the most common gram-positive organism (7%), whereas fungi were isolated in 13% cases. Importantly, 42% of isolates were MDR, whereas 25% were XDR.^290^ If required, was delayed beyond 4 weeks and done primarily employing minimally invasive techniques. The primary outcome measure was independent predictors of in-hospital mortality. Of 209 patients with AP, 81 (39%) gram-positive bacteria including *Staphylococcus aureus, Streptococcus faecalis, Enterococcus* as well as anaerobes, and fungi have also been found.^291,292^ There are several studies that reported increase in the incidence of IPN caused by gram-positive organisms especially in patients who received prophylactic antibiotics for the prevention of development of IPN.^282,293-295^ Garg et al. reported that the majority of the isolates from IPN were sensitive to third-generation cephalosporins and quinolones. A more recent study from India observed that amikacin and imipenem were active against the majority of the gram-negative organisms isolated from IPN.^279,287^ Resistance in gram-negative organisms to aminoglycosides, quinolones, beta-lactam/beta-lactamase inhibitors as well as to carbapenems has increased over last few decades. However, they remain sensitive to colistin and tigecycline. Gram-positive organisms remained sensitive to vancomycin, linezolid, and teicoplanin.

#### Evidence Statement

Gram-negative organisms are the most common organisms isolated from infected pancreatic necrosis following acute pancreatitis in Indian patients. Prophylactic antibiotic use in patients of AP to prevent IPN has been associated with increased risk of infection with gram-positive organisms. Resistance to carbapenems, beta-lactam/beta-lactamase inhibitors and quinolones in gram-negative organisms isolated from IPN has increased, however, with maintaining sensitivity to colistin and tigecycline.

### What are the Empirical Antibiotics of Choice for Treatment of Pancreatic Infection Following Acute Pancreatitis?

Initial reports on use of prophylactic antibiotics in patients with AP to prevent IPN was associated with a reduction in the incidence of IPN and mortality, however, well-designed RCTs and meta-analysis failed to confirm the advantage of prophylactic antibiotics.^280,296,297^ Antibiotics should be prescribed in patients with evidence of IPN (positive image-guided FNA or surgical specimen) or suggested by presence of air within the necrotic pancreatic tissue or persistent fever with leucocytosis and multiorgan failure.^277,278^ Empirical antibiotic regimen is selected based upon the local susceptibility pattern, pharmacokinetic properties of antibiotics and previous antibiotic exposure. Gram-negative organisms isolated from IPN show varying susceptibility to aminoglycosides, cephalosporins, quinolones, piperacillin-tazobactam, and carbapenems. Over the past few decades, there is an increase in the resistance among GNBs isolated from IPN to cephalosporins, quinolones, piperacillin-tazobactam and carbapenems with maintained sensitivity to colistin. Various pharmacokinetic studies have demonstrated the existence of blood pancreatic barrier and this barrier is responsible for the selective uptake of antibiotic drugs into the pancreas.^298,299^ These studies demonstrate that carbapenems have the highest while as aminoglycosides have the least penetration to pancreatic tissue.^299^

Duration of antibiotic therapy in patients with IPN is not clear. However, Malaysian Society of Intensive Care suggests that duration should be guided by a serial assessment of clinical and radiological response.^300^ There are multiple case series, observational studies, and meta-analysis which suggest that conservative management with the use of antibiotics in patients with IPN is associated with improved outcome and less mortality as compared to surgical debridement.^301-305^ Percutaneous drainage or endoscopic necrosectomy should be considered if the patient fails to improve or deteriorates clinically.^277,278^

#### Evidence Statement

Prophylactic use of antibiotics in patients with necrotizing pancreatitis has not been shown to reduce the incidence of pancreatic infection and mortality. Presence of persistent fever, leucocytosis, multiorgan failure and presence of air within pancreatic necrosis suggest infected pancreatic necrosis. Cephalosporins, piperacillin-tazobactam, quinolones, and carbapenems have the highest whereas aminoglycosides have the lowest penetration into necrotic pancreatic tissue. Response to antibiotic therapy is assessed by clinical and radiological parameters.

#### Recommendations

- Routine use of prophylactic antibiotics to prevent pancreatic infection following acute pancreatitis of any severity is not recommended (1A).
- Empirical antibiotic regimen in patients with infected pancreatic necrosis should be guided by local microbiological data, susceptibility pattern, the pharmacokinetic property of antibiotics and previous antibiotic exposure (UPP).
- In treatment-naïve patients with evidence of infected pancreatic necrosis, we recommend empirical treatment with either carbapenems, piperacillin-tazobactam or cefoperazone- sulbactam (2A).
- In patients not responding or already exposed to the piperacillin-tazobactam, cefoperazone- sulbactam or carbapenems, colistin should be added to the empirical regime (3B).
- Duration of antibiotic therapy should be guided by clinical, radiological and laboratory parameters (UPP).
- Patients not responding to antibiotics should undergo necrosectomy and drainage (3B).

## BILIARY SEPSIS

### Acute Cholangitis

Acute cholangitis (AC) is a bacterial infection of the biliary tract that commonly occurs in an obstructed system and leads to systemic signs of infection. Choledocholithiasis is the leading cause of acute cholangitis.^306^ AC is classified as mild, moderate and severe based on organ dysfunction and various biochemical abnormalities.^307^ Grade III AC is associated with organ dysfunction that includes any of the following: hypotension requiring either inotropic or vasopressors, confusion, PaO_2_: FiO_2_ ratio <300, serum creatinine levels> 2 mg/dL, an international normalized ratio >1.5 or platelet counts <100 × 10^9^/L. Grade II cholangitis is associated with any two of the following conditions: WBC count >12,000/mm^3^ or <4,000/mm^3^, high fever (≥39° C), age >75 years, hyperbilirubinemia (>5 mg/dL) or hypoalbuminemia. Grade I do not meet any of the grade III or grade II criteria. Management of acute cholangitis depends on the severity of the illness and include administration of antibiotics and biliary drainage to relieve the obstruction. Drainage can be done selectively in patients with mild cholangitis, within 24–48 hours in patients with moderate cholangitis and immediately in case of severe cholangitis.^308^

### What is the Incidence, Risk Factors and Microbiology of Biliary Infection in ICU? What are the Empirical Antibiotics of Choice for Treatment of Biliary Infections in ICU?

#### Incidence and Risk Factors

The incidence of acute cholangitis varies with underlying etiology. In patients with cholelithiasis symptomatic acute cholangitis develops in 0.2–9% of cases.^309,310^ The incidence of acute cholangitis after endoscopic retrograde cholangiopancreatography (ERCP) ranges from 0.4 to 10%.^311,312^ Risk factors for acute cholangitis include obstruction of the biliary tree (choledocholithiasis, biliary stricture, cholangiocarcinoma, periampullary carcinoma, stent placement for biliary drainage or worm infestation) or biliary intervention (ERCP, post-surgical biliary stricture).^313–317^

#### Evidence Statement

Incidence of acute cholangitis varies with underlying etiology and ranges from 0.2 to 10%. Cholelithiasis, choledocholithiasis, benign and malignant common bile duct (CBD) strictures, CBD interventions and stenting are the most common risk factors for cholangitis.

### Microbiology of Acute Cholangitis

Various observational studies among patients with acute cholangitis from India and across the world have reported that gram-negative enteric organisms are the most common pathogens isolated from bile and/or blood.^315,318-322^ In patients with nosocomial acute cholangitis e.g., postoperative state, with indwelling biliary stents or those with malignant biliary obstruction, more resistant organisms such as MRSA, VRE, and *Pseudomonas* are frequently detected as causative microorganisms. Risk factors for MDR organisms causing acute cholangitis include previous hospitalization and antibiotic use within 90 days.^317^Although the bacteriological profile of acute cholangitis has remained stable over the last few decades, their antibiotic susceptibility pattern has changed. Most of the gram-negative isolates show varying sensitivity to carbapenems, piperacillin-tazobactam, cefoperazone-sulbactam, aminoglycosides and quinolones, with increased resistance to cephalosporins and penicillins.^315,317,319–322^

#### Evidence Statement

Gram-negative organisms are the most common organisms isolated from patients with acute cholangitis. Most of the pathogens isolated are susceptible to third generation cephalosporins (such as cefoperazone-sulbactam), aminoglycosides, quinolones, ureidopenicillins and carbapenems. Risk factors for multidrug drug resistance organisms causing acute cholangitis include indwelling biliary stent, malignant biliary obstruction, previous hospitalization and antibiotic use within 90 days.

### What is the Empirical Antibiotic Regimen for Acute Cholangitis?

Empirical antibiotic regimen in patients with acute cholangitis depends on the antimicrobial activity against causative bacteria, severity of cholangitis, past history of antimicrobial administration to the patient, local susceptibility patterns (antibiogram) of the suspected causative organisms and biliary penetration of the antimicrobial agents.^323^ Biliary obstruction reduces the antibiotic concentration within the bile and improves after biliary drainage, therefore should be considered in all patients of acute cholangitis.^308^ Tokyo guidelines for management of acute cholangitis suggest monotherapy with beta-lactam/beta lactamase inhibitor (cefoperazone-sulbactam, piperacillin-tazobactam) or carbapenems or fluoroquinolone plus metronidazole to cover anaerobes.^324^ IDSA suggests combination of beta-lactam/beta lactamase inhibitor (BL/BLI) or carbapenems or quinolones with metronidazole for moderate to severe community acquired cholangitis. For nosocomial moderate to severe cholangitis combination of BL/BLIs or carbapenems or quinolones with metronidazole plus vancomycin is advised.^325^ IDSA suggests that antimicrobial therapy of established infection should be limited to 4–7 days, unless it is difficult to achieve adequate source control.^325^ Previous Tokyo guidelines recommended antibiotics for 2–3 days in case of mild and 5–7 days in case of moderate to severe cholangitis.^324^ However, latest revised Tokyo guidelines for management of acute cholangitis suggest duration of antibiotic to be 4–7 days once the source of infection is controlled.^323^ Duration of antibiotics may be guided by clinical response.

#### Evidence Statement

Empirical antibiotic regime in patients with acute cholangitis is guided by the severity of the disease, local antibiotic susceptibility pattern and biliary penetration of the antibiotics. Duration of antibiotics depends on the severity of cholangitis and adequacy of source control. Biliary drainage (percutaneous or endoscopic) is required in addition to antibiotic use in management of acute cholangitis.

#### Recommendations

- Empirical antibiotic therapy should be guided by severity of the cholangitis, local microbiological susceptibility patterns, biliary penetration of antibiotics and previous antibiotic exposure (UPP).
- We recommend either beta-lactam/beta-lactamase inhibitor (such as cefoperazone-sulbactam or piperacillin/tazobactam) or carbapenems (imipenem/meropenem) as monotherapy in patients with moderate to severe cholangitis (3B).
- We recommend antibiotic duration for 4–7 days in patients of acute cholangitis after adequate source control (2B).
- Biliary drainage should be considered in all patients with cholangitis in addition to empirical antibiotic therapy. (1A)

### Liver Abscess

Liver abscess is an infectious, space-occupying lesion in the liver. Pyogenic and amoebic liver abscess are the two most common causes of liver abscess. Appropriate initiation of antibiotics will help to prevent potentially lethal complications like bacteremia and spread of abscess to other organs.

#### Incidence and Risk Factors

The incidence of pyogenic liver abscess varies from as low as 2.3 per lac population to as high as 446 per lac depending upon the presence of risk factors which predispose the person to liver abscess.^326,327^ The various risk factors for pyogenic liver abscess include male gender, older age, diabetes mellitus, biliary diseases, endobiliary procedures, alcoholism, hepatobiliary malignancies, and infected cystic liver lesions.^327-331^ and early diagnosis may be difficult.

### What are the Most Common Organisms Causing Liver Abscess in ICU?

Microorganisms causing liver abscess have shown varying trends over the years. The earlier studies had shown predominantly gram-positive organisms like *Streptococcus* as common cause of pyogenic liver abscess.^332^ However, the recent studies have reported gram-negative organisms (including *Klebsiella pneumoniae, E. coli, P. aeruginosa*) to be responsible for majority of cases of pyogenic liver abscess.^328,329,333–337^ Rarely pyogenic liver abscess is caused by organisms like *Burkholderia, Prevotella* and anaerobic bacteria including *eikenella* and *Peptostreptococcus*.^338,339^ In Indian setting, amoebic liver abscess is the most common cause of liver abscess caused by infection with *Entamoeba histolytica*.^340^

#### Evidence Statement

Amoebic liver abscess is the most common cause of liver abscess in Indian setup. The incidence of pyogenic liver abscess varies from 2.3 to 446 per 100000 hospital admissions per year. Gram-negative organisms (*E. coli* and *Klebsiella*) are the most common organisms causing pyogenic liver abscess. Risk factors for pyogenic liver abscess include diabetes mellitus, older age, male gender, biliary diseases, biliary procedures, alcoholism, malignancy, intra-abdominal infection, and cystic lesions in the liver.

### What are the Empirical Antibiotics of Choice for Treating Liver Abscess in ICU?

### Amebic Liver Abscess

Empirical treatment of amoebic liver abscess consists of a combination of tissue agent and a luminal agent. Metronidazole is the drug of choice for management of amoebic liver abscess. Metronidazole given for a period of 10 days has been shown to be effective.^341^Alternatives to metronidazole include tinidazole, ornidazole, and nitazoxanide.^342,343^ The luminal agents used to remove any intraluminal cysts include paromomycin, diiodohydroxyquin or diloxanide, even if the stool microscopy is negative. Routine use of drainage of amoebic liver abscess is not indicated in uncomplicated cases.^341^ However, addition of needle aspiration to metronidazole has shown to hasten clinical improvement especially in large abscess (5–10 cm).^337^ Surgical intervention is required ifthere is noresponse to medical management.^341,344^

#### Evidence Statement

Metronidazole is the drug of choice for treatment of amoebic liver abscess. The optimum duration of treatment in patients with amoebic liver abscess is 10–14 days. Routine needle aspiration of amoebic liver abscess is controversial. Addition of aspiration to drug therapy in patients with amoebic liver abscess of >5 cm in size hastens clinical improvement.

#### Recommendations

- We recommend metronidazole as an initial antibiotic of choice in patients with amoebic liver abscess (2A).
- We recommend antibiotic treatment for a period of 10–14 days in patients with amebic liver abscess (3B).
- Needle aspiration of amoebic liver abscess is recommended in patients with lack of clinical improvement in 48–72 hours, left lobe abscess, abscess more than 5–10 cm or thin rim of liver tissue around the abscess (<10 mm) (UPP).

### Pyogenic Liver Abscess

Antibiotics that are effective in treatment of pyogenic liver abscess include third and fourth generation cephalosporins (such as ceftriaxone, cefepime), aminoglycosides, fluoroquinolones, beta-lactam/beta-lactamase inhibitor (piperacillin-tazobactam), carbapenems, and metronidazole.^334,345-347^ Carbapenems are effective for treatment of liver abscess caused by melioidosis or infection with ESBL producing organism.^348,349^

Empirical regimen should include a broad-spectrum parenteral antibiotic pending microbiologic analysis of the abscess contents. It should cover enteric gram-negative bacilli, streptococci, and anaerobes. Antibiotic therapy should generally be continued for four to six weeks.^335^ However, the optimal duration of therapy is unclear and is guided by clinical and radiological response. Studies have reported that shorter courses of antibiotics for 2–4 weeks are effective as well.^335,345,350^ In case of abscess cavity with a size less than 5 cm, a needle aspiration is preferred and in case of abscesses more than 5 cm in size, percutaneous catheter drainage is preferred.^351–353^ Surgical drainage is required in cases of abscesses with viscous contents obstructing the catheter, underlying disease requiring primary surgical management and inadequate response to percutaneous drainage within 7 days.^354^

#### Evidence Statement

Beta-lactam/beta-lactamase inhibitors, metronidazole and carbapenems are effective antibiotics for management of pyogenic liver abscess. Carbapenems are effective in case of suspected infection with ESBL producing organisms or melioidosis. Antibiotics are required for prolonged periods ranging from 2–4 weeks. Clinical and radiological assessment is required to guide the adequate treatment duration.

#### Recommendations

- We recommend beta lactam/beta lactamase inhibitors with metronidazole in patients with pyogenic liver abscess for a duration of 2–4 weeks (2A).
- We recommend carbapenems in case of infection with ESBL producing organisms or melioidosis (2B).

### Peritonitis

Peritonitis is defined as an inflammation of the peritoneum from any cause. Peritonitis occurs due to a variety of etiologies, of which the most common is infections. It is broadly classified as primary, secondary and tertiary. Primary peritonitis, also known as spontaneous bacterial peritonitis (SBP), has no identifiable anatomical dehiscence. It is usually managed non-surgically. The risk factors for development of primary peritonitis include advanced cirrhosis, nephrotic syndrome and peritoneal dialysis.^355,356^ Secondary peritonitis is the infection of peritoneum that occurs in critical ill patients secondary to organ perforation, anastomotic leak or trauma to the gastrointestinal tract. Tertiary peritonitis may be defined as a severe recurrent or persistent intra-abdominal infection after apparently successful and adequate surgical source control of secondary peritonitis.^357^ It leads to prolonged systemic inflammation and is usually associated with high mortality (30–64%). Longer ICU stay, emergency abdominal surgery and total parenteral nutrition are risk factors associated with the development of tertiary peritonitis.^358-363^

### What are the Most Common Organisms Causing Peritonitis in ICU?

Enteric gram-negative organisms including *E. coli, Klebsiella* and Enterobacteriaceae are the most common causative agents for primary and secondary peritonitis.^364,365^ Other organisms include gram-positive bacteria (such as *Enterococcus*) as well as anaerobes (i.e. *Bacteroides*).^365^ Tertiary peritonitis is usually due to opportunistic and nosocomial drug resistant bacteria and fungi. Various organisms reported are *Enterococcus, Candida, Staphylococcus* and *Enterobacter*.^363,366^

#### Evidence Statement

The risk factors for development of primary peritonitis are decompensated cirrhosis, nephrotic syndrome and peritoneal dialysis. The risk factors for development of secondary peritonitis include intra-abdominal organ perforation, post intra-abdominal surgery, and trauma. Longer ICU stay, urgent operation on hospital admission, total parenteral nutrition and stomach-duodenum as primary infection site are associated with the development of tertiary peritonitis. Gam-negative enteric organisms are the common causes of primary and secondary peritonitis. Other organisms include gram-positive as well as anaerobic bacteria. The organisms commonly isolated in tertiary peritonitis are *Candida, Enterococcus faecium* and *Staphylococcus epidermidis*.

### What are the Empirical Antibiotics of Choice for Treating Peritonitis in ICU?

### Primary Peritonitis

Cephalosporins and fluoroquinolones are effective against majority of the cases of primary peritonitis.^364,367-370^ Antibiotics for a period of 7–10 days are effective in SBP.^364,367^ In difficult to treat SBP, cefepime and imipenem are reported to be effective.^371^

### Secondary Peritonitis

The antibiotics effective in secondary peritonitis are beta lactam/beta lactamase inhibitors (piperacillin-tazobactam), quinolones, carbapenems, aminoglycosides and metronidazole.^365,372,373^ When enterococci are considered, addition of vancomycin or linezolid is required for a spectrum adequacy rate of more than 95%.^374^ The average duration of antibiotic therapy is 10 to 14 days. However, recently the emphasis is on a shorter course of antibiotics after adequate source control. The recent STOP –IT trial has found that in patients after an adequate source control, outcomes after fixed-duration antibiotic (approximately 4 days) were similar to those after a longer course of antibiotics (approximately 8 days).^375^

#### Evidence Statement

Third generation cephalosporins are the most effective antibiotic therapy for primary peritonitis. Antibiotics are usually required for 7–10 days for adequate treatment. Most of the organisms isolated in secondary peritonitis are sensitive to beta lactam/beta lactamase inhibitors or carbapenems. For gram-positive organisms, vancomycin and linezolid are effective treatment options. Short duration of antibiotic treatment (4 days) are as effective as longer duration after an adequate source control.

#### Recommendations

- We recommend third generation cephalosporins (such as cefotaxime and ceftriaxone) for a duration of 7–10 days in patients with primary peritonitis (2A).
- We recommend either beta-lactam/beta-lactamase inhibitor or carbapenems with an anaerobic cover (using metronidazole) for the treatment of secondary peritonitis (2A).
- For secondary peritonitis antibiotic treatment is required for 4 days after an adequate source control(2A).

### CNS Infections in ICU

Infections of central nervous system (CNS), either community or hospital acquired, are frequent causes of admission to ICU. Bacterial meningitis and brain abscess are one of the commonest CNS infections and can result in significant morbidity and mortality. CNS infections are markedly different from systemic infections because of closed anatomic space and immunologic isolation of CNS from the rest of the body. They often have nonspecific clinical manifestations posing a diagnostic challenge to the clinician. Early suspicion, rapid diagnosis and aggressive management are essential for better outcomes and to prevent various complications and neurological sequalae.

### What are the Most Common Organisms Causing Acute Bacterial Meningitis in ICU?

Bacterial meningitis, an infection of meninges and subarachnoid space, is a complex disorder in which injury is caused partly by the causative organism and partly by the host inflammatory response. Bacterial meningitis is a medical emergency, given the associated mortality and neurological sequalae requiring prompt recognition, rapid diagnostic evaluation and emergent antimicrobial therapy. Hence accurate information regarding incidence, risk factors and microbiological profile of bacterial meningitis is necessary to ensure appropriate empirical antibiotic management. Meningitis can be community acquired or associated with a variety of neurosurgical procedures (e.g., craniotomy, placement of invasive neuro-monitoring techniques, external ventricular drain catheters or cerebrospinal fluid shunts) and penetrating head injury. The latter group is classified as nosocomial meningitis or healthcare associated meningitis and ventriculitis. Both groups differ in their pathogenic mechanisms, risk factors, etiological agents microbial susceptibility patterns and hence are discussed separately.

### Community-acquired Meningitis

The incidence of bacterial meningitis in USA was 2 cases per 100,000 population in 1998–1999 that decreased to 1.38 cases per 100,000 population in 2006–2007; most common organisms were *Streptococcus pneumoniae* (56.8%), *Neisseria meningitidis* (17.2%), group B streptococci (16.7%), *Hemophilus influenzae* (5.8%) and *Listeria monocytogenes* (3.2%).^376^ In a retrospective study of 195 culture positive acute bacterial meningitis patients, most common organism was *Streptococcus pneumoniae* followed by *Staphylococcus aureus* and *Klebsiella pneumoniae*.^377^ Various large studies have found *S. pneumoniae* as the most common etiological agent followed by *N. meningitidis, L. monocytogenes, H. influenzae* and group *B Streptococcus*,^378–382^ though *S. aureus* has also been reported as one of the commonetiological agents by some.^379,380^ Otitis media, immunocompromised status, elderly population and prior use of antibiotics have been described as risk factors for bacterial meningitis.^378,383,384^Various Indian studies have yielded similar results.^385–388^

#### Evidence Statement

The incidence of community acquired pyogenic meningitis ranges from 2 to 7.40 per lakh population. The common causative organisms include *Streptococcus pneumoniae, Neisseria meningitidis*, other streptococci, *Hemophilus influenzae* and *listeria monocytogenes*. Other causative organisms are *Staphylococcus* species, gram negative bacilli, *Pseudomonas* and *Acinetobacter*. Common risk factors for community acquired bacterial meningitis are otitis media, elderly population, depressed immune status and prior use of antibiotics.

### Nosocomial Meningitis

Nosocomial meningitis may result from various invasive procedures including craniotomy, placement of internal or external ventricular catheters, lumbar puncture, intrathecal infusions of medications, spinal anesthesia or complicated head trauma or rarely from metastatic infection in patients with hospital-acquired bacteremia.

Incidence of post ventricular drain or catheter related infections has been studied in many retrospective and prospective studies and ranges from 5.6 to 14.2% and 5.5 to 19%, respectively.^389-394^ A systematic review from January 1990 through March 2008 reporting on ventriculostomy and extra-ventricular drain (EVD) related CNS infections described incidence of 2–27%.^395^ *Staphylococcus epidermidis* (70%) is the most common microbiological agent followed by gram-negative bacilli (15%) and *Staphylococcus aureus* (10%). Risk factors described included EVD duration greater than 11 days, frequency of cerebrospinal fluid (CSF) sampling, intraventricular hemorrhage and surgical technique (subcutaneously tunneled EVD, Rickham reservoir with percutaneous CSF drainage). Post craniotomy or neurosurgery incidence of meningitis ranges from 0.02 to 9.5%.^391,396-402^ Most of the studies have reported *Staphylococcus* to be the most common causative organism.^391,396,398,400,401^ Few studies have also reported *Acinetobacter* and Enterobacteriaceae as the most common organisms.^397,399^ Postoperative CSF leak has been consistently shown to be a risk factor.^391,396–398,400,401,403^ Other risk factors are placement of external shunts, longer duration of drainage, multiple intracranial operations, emergency or prolonged surgery, diabetes and elderly population.^391,396-401^ The role of prophylactic antibiotics for post neurosurgery and craniotomy meningitis had been debatable, however, a recent meta-analysis of 7 RCTs including 2365 postcraniotomy patients found that prophylactic antibiotic use reduced the rate of post neurosurgical meningitis.^404^ The incidence of post spinal blockade meningitis is very low with a large retrospective analysis of 12,60,000 spinal blockades and 450,000 epidural blockades showing incidence to be 1 in 53000 with alpha-hemolytic streptococci as the most common causative organism.^405^ Exogenous inoculation is a risk factor and various measures such as hand disinfection, sterile gloves, face masks and operating caps decrease the risk of development of meningitis.^406^ The incidence of meningitis or ventriculitis in patients with post traumatic head injury is 1.39–2%.^407,408^ Common organisms include CONS, gram negative bacilli and *Acinetobacter*. Lumbar and ventricular drains are described as the risk factors. A recent Cochrane systematic review has not shown benefit of using prophylactic antibiotics in patients with basilar skull fracture, independent of CSF leakage.^409^ Post internal ventricular drain infections incidence has been reported between 5.9% and 15.2% in various prospective and retrospective studies. Most common causative organisms included *Staphylococcus aureus* and CONS.^410,411^ Postoperative CSF leak, use of single gloves and number of times shunt system exposed to breached surgical gloves were described as risk factors.^412^

#### Evidence Statement

Incidence of post ventricular drain or catheter meningitis ranges from 2 to 27%. Commonly implicated organisms are CONS (especially *Staphylococcus epidermidis*), *Staphylococcus aureus, Acinetobacter, Pseudomonas* and Enterobacteriaceae. Risk factors are repeated catheterization, higher catheter duration, CSF sampling, presence of concomitant systemic infection and surgical technique i.e., subcutaneously tunnelledextraventricular drain (EVD), Rickham reservoir with percutaneous CSF drainage. Incidence of post craniotomy or post neurosurgery meningitis is 0.02 to 9.5%. Most commonly implicated organisms are *Staphylococcus aureus*, coagulase-negative staphylococci (especially *S. epidermidis*), Enterobacteriaceae, *Acinetobacter* and *Pseudomonas*. Risk factors include CSF leak, EVD, longer duration of drainage, multiple operations, lack of antibiotic prophylaxis and emergency surgery. Incidence of post-neuroaxial blockade meningitis is 0.2 per 10000 with viridans streptococci and *Staphylococcus aureus* being common organisms. Exogenous inoculation is the main risk factor. Post head trauma meningitis incidence ranges from 1.39 to 2% with CONS, *Acinetobacter* and Enterobacteriaceae as common microbes and prolonged hospitalization, insertion of lumbar and ventricular drain as common risk factors. Post internal ventricular drain infection incidence ranges from 5.9 to 15.2%. Most common causative organisms are CONS, *Staphylococcus aureus*, gram negative bacilli, group D streptococci and *Propionibacterium acnes*. CSF leak, single gloves use and number of times shunt exposed to breached surgical gloves are the risk factors.

### What are the Empirical Antibiotics of Choice for Treating Acute Bacterial Meningitis in ICU? What should be the Duration of Antibiotic Treatment?

Early diagnosis and urgent appropriate antimicrobial therapy along with other adjunctive therapy is necessary to reduce morbidity and mortality associated with bacterial meningitis. As isolation of microorganism takes time and sometimes it may not be isolated at all, empirical antimicrobial therapy need to be based on most likely involved organism as determined by presence of risk factors for various organisms and local antibiotic susceptibility pattern.

### Community-acquired Meningitis

The evidence regarding empirical antibiotic choice in acute bacterial meningitis (ABM) is limited. A retrospective study found reduced penicillin susceptibility in 23% patients with meningitis, including 16% in community acquired meningitis. Ceftriaxone combined with penicillin was found adequate in 97% cases.^413^ Retrospective study by Erdem et al. reported inadequacy of ceftriaxone alone in treatment of pneumococcal meningitis in view of increasing penicillin resistance in pneumococci worldwide.^414^ A Cochrane review in 2007 comparing third generation cephalosporins (ceftriaxone or cefotaxime) with conventional antibiotics (ampicillin-chloramphenicol combination, or chloramphenicol alone) as empirical therapy for ABM in adults and children found no statistically significant difference between the groups in the risk of death, risk of deafness or risk of treatment failure although significantly decreased chances of culture positivity of CSF after 10–48 hours with the third generation cephalosporins at the cost of increased risk of diarrhea.^415^ A recent Indian study including 266 culture positive ABM patients (including 142 CAM patients) found that gram positive pathogens exhibited maximum sensitivity to vancomycin and linezolid whereas most gram-negative pathogens were sensitive to carbapenems.^416^ Seven days antibiotic therapy has been recommended for *N. meningitidis* and *H. influenzae*, 10–14 days for *S. pneumoniae*, 14–21 days for *S. agalactiae*, 21 days for aerobic GNB and 21 days or more for *L. monocytogenes*.^417^

#### Evidence Statement

Choice of antibiotics depends on most likely causative microorganism, local antibiotics sensitivity patterns, mechanism of infection and patient's predisposing condition. Most commonly recommended empirical antibiotic regimens include third generation cephalosporin plus vancomycin, third generation cephalosporin monotherapy and penicillin monotherapy. Addition of amoxicillin, ampicillin or benzyl-penicillin has been recommended in patients older than 50 years.

#### Recommendations

- We recommend third generation cephalosporin (preferably ceftriaxone) plus vancomycin as empirical antibiotics of choice for community acquired meningitis (3A).
- We recommend to add ampicillin or amoxicillin if age >50 years (3A).
- If beta-lactams are contraindicated, we recommend chloramphenicol plus vancomycin as antibiotic of choice, and to add cotrimoxazole if age >50 years (3A).
- We recommend ciprofloxacin or aztreonam plus vancomycin as alternative regimen and to add cotrimoxazole, if age greater than 50 years (UPP).
- We recommend duration of antibiotics based on suspected or isolated organisms i.e., 10–14 days for *Streptococcus pneumoniae*, 14–21 days for *Streptococcus* agalactiae, 7 days for *neisseria meningitidis* or *Hemophilus influenzae*, 21 days for aerobic gram-negative bacilli, and 21 days or more for *listeria monocytogenes* (3A).
- If no microorganism is identified, antibiotics should be given for at least 10–14 days (3A).

### Nosocomial Meningitis

Treatment recommendations for nosocomial meningitis are largely based upon expert opinion. IDSA guidelines for management of bacterial meningitis recommend vancomycin plus third generation cephalosporin for post basilar skull fracture meningitis; vancomycin plus cefepime, ceftazidime or merepenemhas been recommended for post neurosurgery nosocomial meningitis or meningitis occurring after CSF shunt or penetrating trauma.^417^

A systematic review of intraventricular or intrathecal use of polymyxins in patients with gram-negative meningitis including 31 case reports and case series found limited available evidence to suggest addition of intraventricular or intrathecal antimicrobials to systemic therapy in gram-negative meningitis. Toxicity was dose-dependent and reversible.^418^Another review for use of intraventricular use of vancomycin found its use safe and effective.^419^ IDSA guidelines recommend vancomycin plus an anti-pseudomonal beta-lactam (such as cefepime, ceftazidime, or meropenem) as empiric antimicrobial of choice for suspected healthcare associated ventriculitis and meningitis.^420^ Regarding optimum duration of antibiotic therapy, IDSA recommends therapy for 10 days if coagulase-negative *Staphylococcus* or *P. acnes* with no or minimal CSF pleocytosis, normal CSF glucose, and few clinical symptoms or systemic features; 10–14 day treatment is recommended in case of significant CSF pleocytosis, CSF hypoglycorrhachia, clinical symptoms or systemic features. Treatment for 21 days is recommended for gram negative bacilli and *Staphylococcus aureus*. In patients with repeatedly positive CSF cultures on appropriate antimicrobial therapy, IDSA recommends treatment to be continued for 10–14 days after the last positive culture.^420^

#### Evidence Statement

Vancomycin in combination with cefepime, ceftazidime or meropenem is commonly recommended empirical antibiotic regimen for nosocomial meningitis. Alternative regimens include third generation cephalosporin or meropenem monotherapy or ceftriaxone plus flucloxacillin or cloxacillin combination therapy. Limited available evidence shows efficacy of intraventricular or intrathecal antibiotics in management of nosocomial meningitis poorly responsive to systemic antibiotics.

#### Recommendations

- We recommend vancomycin plus cefepime or ceftazidime or meropenem as empirical antibiotics of choice for nosocomial meningitis (3A).
- Colistin may be given if incidence of CRE or drug resistant *Acinetobacter* is high in the specific unit (UPP).
- If beta-lactams are contraindicated, we recommend to replace beta-lactam with aztreonam or ciprofloxacin (3A).
- Intraventricular or intrathecal antibiotics should be considered if infection responds poorly to appropriate systemic antibiotics clinically or microbiologically (3A).

### What are the Most Common Organisms Causing Brain Abscess in ICU?

Brain abscess is a serious life-threatening emergency with high morbidity and mortality. The management of brain abscess is challenging and needs good clinical and surgical skills for better outcomes. The choice of pharmacological therapy should be based on the most likely organism, patient's predisposing condition or risk factors, mechanisms of infection, antimicrobial susceptibility patterns and on the ability of the antimicrobial agent to penetrate the abscess.

In a recent single-center retrospective study over 62 years including 620 patients of brain abscess, the incidence of brain abscess (per lakh population) was 2.5 between 1952 to 1972, 2.6 in 1980 to 1991 and 2.2 in 2002 to 2014.^421^ *Staphylococcus aureus* is one of the commonest organism followed by *Proteus* sp. and *Streptococcus*. Chronic ear infection is a common predisposing factor (65% cases).^422^ *Streptococcus* (34%), followed by *Staphylococcus* (18%), gram-negative enteric bacilli (15%), *P. seudomonas* and *Haemophilus* (2% each) were found to be the commonly isolated organisms in a recent meta-analysis. *Peptostreptococcus, Bacteroides*, and *Fusobacterium* were isolated in 3%, 6%, and 2%, respectively and polymicrobial etiology was found in 23% cases.^423^ Most common predisposing condition was otitis media followed by sinusitis, heart disease, post-traumatic, hematogenous, pulmonary disease, postoperative, odontogenic, immunocompromised and meningitis. Two retrospective studies found *Staphylococcus aureus* to be the most common causative organism followed by *Streptococcus*.^424,425^ Otitis media was the most common risk factor followed by congenital heart disease, paranasal sinus infections, dental causes, trauma, and post-operative state.^424-427^ Various prospective Indian studies found streptococci to be most common microbe.^426,427^

#### Evidence Statement

The incidence of brain abscess ranges from 1.3 to 2.6 cases per lakh population. Most commonly involved micro-organism*s* include *Streptococcus* (especially *S. viridans*), *Staphylococcus* (especially *S. aureus*), gram negative bacilli, anaerobes (*Bacteroides, Peptostreptococcus, Fusobacterium*), *Pseudomonas* and *H. influenzae*. Polymicrobial etiology accounts for 23–26% cases. Risk factors include otitis media, sinusitis, head trauma, congenital heart diseases, hematogenous spread, surgery, immunocompromised status, pulmonary disease, meningitis, and odontogenic infections.

### What are the Empirical Antibiotics of Choice for Treating Brain Abscess in ICU? What should be the Duration of Antibiotic Treatment?

The data regarding the efficacy of various empirical antibiotic regimens in the management of brain abscess is limited to observational studies and expert opinion. In a systematic review and meta-analysis of clinical characteristics and outcomes of brain abscess, 17 studies described how many patients received which regimen.^428^ The most common empiric treatment consisted of a third-generation cephalosporin combined with metronidazole, which was given in 53% of cases while vancomycin was added in addition 15% cases. Other regimens had combinations of chloramphenicol, metronidazole with penicillin (9%), ampicillin, gentamicin with metronidazole (9%), and imipenem monotherapy (4%).^428^ There is insufficient evidence to make specific recommendations but on the basis of limited clinical data, recommendations include cefotaxime plus metronidazole with or without rifampicin for post-trauma abscess, linezolid or vancomycin plus rifampicin plus meropenem or piperacillin/tazobactam for post-surgical abscess, cefotaxime or piperacillin-tazobactam plus metronidazole for post middle ear, paranasal sinuses, dental causes and cefotaxime with or without metronidazole or ampicillin-sulbactam for cryptogenic or metastatic abscess. Four to six weeks of antibiotic therapy is required for surgically treated abscess and 6–8 weeks for solely medically treated or multiple surgical abscesses with largest one treated surgically.^429^

#### Evidence Statement

The most common empiric treatment consists of a third-generation cephalosporin combined with metronidazole. Antibiotic duration ranges from 4 to 8 weeks.

#### Recommendations

- We recommend third-generation cephalosporins plus metronidazole as the empirical antibiotic of choice for brain abscess (3A).
- We recommend adding vancomycin if there is a high likelihood of MRSA (3A).
- We recommend vancomycin plus ciprofloxacin if beta-lactams are contraindicated (3A).
- We recommend aztreonam, if ciprofloxacin cannot be given or contraindicated (UPP).
- We recommend minimum 4 weeks of therapy, however duration may be extended according to clinic-radiological response irrespective of aspiration or excision of abscess (3A).

### Skin- and Soft-tissue Infections in ICU

An inflammatory microbial invasion of the epidermis, dermis and subcutaneous tissues is defined as skin and soft tissue infection (SSTI). In ICU, 4.3–10.5% of septic episodes may be caused by SSTIs,^430^ with attributable mortality of 11.7%.^431^ Spectrum of SSTI includes abscess, carbuncle, cellulitis, surgical site infection, diabetic foot, and necrotizing fasciitis. SSTI has been classified based on signs of sepsis and comorbidities. SSTI without any signs or symptoms of systemic toxicity or comorbidities is termed class 1. SSTI in patients with significant comorbidities (diabetes or obesity), but without any evidence of sepsis is termed class 2. Class 3 SSTI refers to SSTI with fever, tachycardia and tachypnea with or without hypotension. Class 4 SSTI refers to life-threatening infections like necrotizing fasciitis along with sepsis.^432^ For treatment decision, it is important to classify SSTIs into purulent (carbuncle, furuncle, and abscess) and non-purulent (necrotizing fascütis, cellulitis, and erysipela). Non-purulent SSTIs are classified into mild (no focus of purulence), moderate (presence of systemic inflammatory response syndrome, i.e., SIRS) and severe (failed oral antibiotics, SIRS, immunocompromised, deeper infection or organ dysfunction). Purulent SSTIs are classified into mild (no systemic signs of infection), moderate (SIRS present) and severe (SIRS along with treatment failure, or organ dysfunction).^433^

### What are the Most Common Organisms and Risk Factors for SSTI in ICU?

*Staphylococcus aureus* (20.9–38.1%) and gram-negative bacilli (29.1–57.4%)have been commonly implicated in SSTIs in India.^434-436^ *Pseudomonas* (11.8–57.4%) and *E. coli* (17.3%) are most common GNBs.^435,436^ High proportion of *Staphylococcus aureus* (40–74%) have been reported to be methicillin-resistant,^435,437^ whereas the majority of (66.7–74%) GNBs have been reported to be ESBL producing.^435^ Necrotizing fascütis is caused mostly by *Streptococcus pyogenes* in monomicrobial form. Clostridial species are also responsible for monomicrobial necrotizing fasciitis.^438^ In polymicrobial necrotizing fascütis, the most commonly implicated pathogens are coliforms, anaerobic bacteria and *Staphylococcus*.^439,440^ Old age, obesity, diabetes mellitus, malignancy, higher APACHE score, longer ICU stay, end-stage renal disease, cirrhosis of the liver, intravenous drug abuse, and neutropenia are risk factors for SSTI.^432,441,442^

#### Evidence Statement

Older age, diabetes mellitus, obesity, malignancy, cirrhosis, and longer ICU stay are risk factors for SSTIs. Gram-positive organisms (*Staphylococcus aureus*) are the most common organism responsible for the SSTIs. *E. coli* and *Pseudomonas* are common pathogens among gram-negative organisms. MRSA and ESBL producing gram-negative organisms are the most common causative agents for SSTIs in ICU. Monomicrobial necrotizing fasciitis is commonly caused by *Streptococcus pyogenes*; mixed coliforms, anaerobes, and *staphylococci* are common causes of polymicrobial necrotizing fasciitis.

### What are the Empirical Antibiotics of Choice for Treating SSTI in ICU? For Empirical Therapy, should Combination Therapy be Preferred over Monotherapy?

Studies on SSTIs specific to ICU settings are not available. A meta-analysis performed by Rebecca et al.^443^ showed the clear superiority of linezolid and vancomycin in treating skin and soft tissue infection caused by *S. aureus*. Teicoplanin is also a good choice for treating severe SSTI caused by MRSA, with similar efficacy and fewer adverse effects as compared to vancomycin.^444-446^ Daptomycin has been shown to have a more rapid clinical cure, reduced the length of hospital stay and lower cost as compared to vancomycin in a prospective study of SSTIs in ICU.^447^ Other RCTs have demonstrated non-inferiority of daptomycin to vancomycin.^448^ MRSA remains sensitive to vancomycin and linezolid, and the majority remain sensitive to clindamycin also (79%).^437^ For gram-negative pathogens, piperacillin-tazobactam and imipenem have been reported to be most effective antibiotics.^430^

#### Evidence Statement

Vancomycin, teicoplanin, daptomycin, and linezolid are effective in SSTIs caused by MRSA. Piperacillin-tazobactam and carbapenems are the most effective antibiotics for ESBL producing gram-negative organisms. Penicillin plus clindamycin are most effective antibiotics in monomicrobial necrotizing fasciitis, whereas a combination of piperacillin-tazobactam, fluoroquinolone and clindamycin are effective for polymicrobial necrotizing fasciitis.

#### Recommendations

- For moderate non-purulent SSTI, we recommend intravenous penicillin or clindamycin as the first choice of antibiotics (2A).
- Severe non-purulent SSTI should be treated with a combination of piperacillin-tazobactam along with coverage for MRSA (vancomycin, teicoplanin, daptomycin or linezolid) (2A).
- Concomitant surgical inspection or debridement should be considered for severe non-purulent SSTIs (2A).
- For severe purulent SSTI, incision and drainage followed by empiric antibiotics including piperacillin-tazobactam, along with MRSA coverage (vancomycin, teicoplanin, daptomycin or linezolid) are recommended (3A).
- Penicillin plus clindamycin is recommended for monomicrobial necrotizing infection caused by *Streptococcus pyogenes* or clostridial species. For polymicrobial necrotizing fasciitis, a combination of piperacillin-tazobactam, fluoroquinolone and clindamycin is recommended (3A).

### What should be the Duration of Antibiotic Treatment for SSTI?

There is limited literature to guide treatment of severe or complicated SSTIs. In uncomplicated SSTI, antimicrobial administration for 5 days was equally effective to 10-day treatment.^449^ Complicated SSTIS may require longer treatment.

#### Evidence Statement

A shorter course of antibiotic therapy is adequate for uncomplicated SSTIs while complicated SSTIs require a longer duration of antibiotic therapy.

#### Recommendations

- Severe nonpurulent SSTIs should be treated with at least 5 days of antibiotics (3A).
- Severe SSTIs with organ dysfunction should be treated with a prolonged course of antibiotics of 2–3 weeks duration (3A).

### Sepsis of Unknown Cause in ICU

Mortality from severe sepsis and septic shock remains consistently high.^450,451^ Delay in antimicrobial therapy is associated with increased in-hospital and overall mortality in severe sepsis and septic shock.^452,453^ Adequate source control, appropriate antibiotic therapy, and organ support are cornerstone for the success in the treatment of patients with sepsis. Delay in the initiation of appropriate antibiotic therapy has been recognized as a risk factor for mortality. While every effort should be made to secure site-specific cultures to guide microorganism-specific therapy, this should never delay the administration of empiric antimicrobials.^454^ Intensive efforts, including imaging, should be undertaken in an attempt to evaluate the source of infection. Two sets of blood cultures and other appropriate microbiological specimens should preferably be taken before empirical therapy. Urgent empirical broad-spectrum coverage to include all common pathogens should be administered.^454^

### What is the Empirical Treatment for Sepsis of Unknown Cause in ICU?

There is a paucity of data on empirical antimicrobial therapy in sepsis of unknown cause in ICU. Combination antimicrobial therapy (using two drugs from a different class) improves survival and clinical outcomes in patients with sepsis who are critically ill and in septic shock as compared to monotherapy.^455^ Beta-lactams with aminoglycosides or fluoroquinolones gives a broad empirical coverage. If the patient has risk factors for MRSA, vancomycin should be added to the regimen.^456^ Accordingly if risk factors for MDR pathogens are present in an individual patient, beta-lactam of choice is a carbapenem. In India, empirical therapy should cover for various tropical infections till a definite diagnosis is reached. Third-generation cephalosporins with doxycycline is an appropriate option keeping this fact in mind.

#### Evidence Statement

Empirical therapy with dual class (with different mechanisms of action) combination antimicrobial therapy for sepsis of unknown cause in ICU is associated with have better clinical outcomes. Empirical therapy with either piperacillin-tazobactam or carbapenems in combination with aminoglycosideor fluoroquinolone has been shown to give appropriate broad coverage leading to better clinical outcomes as compared to monotherapy.

#### Recommendations

- We recommend empirical antimicrobial therapy with a combination of ceftriaxone and doxycycline or a macrolide for community-acquired sepsis of unknown origin in ICU (UPP).
- We recommend empirical antimicrobial therapy with a combination of beta-lactam/beta-lactamase inhibitor and fluoroquinolone or aminoglycoside for nosocomial sepsis of unknown origin in ICU (UPP).
- Empiric therapy should attempt to provide antimicrobial activity against the most likely pathogens based upon clinical features along with local patterns of infection and resistance (UPP).
- Duration of therapy is 7–10 days, though longer courses may be appropriate in patients with a slow response (3B).

### Empirical Antifungals for Non-neutropenic Patients in ICU

Invasive fungal infection (IFI) is an important cause of morbidity and mortality among critically ill patients. Early institution of antifungal therapy is pivotal for mortality reduction. Starting targeted antifungal therapy after culture positivity or identification of pathogen requires a long time. Therefore, alternative strategies (defined as untargeted antifungal treatment) for antifungal therapy institution in patients without proven microbiological evidence of fungal infections have been considered.^457^ Untargeted antifungal strategies include prophylactic antifungals, preemptive antifungals, and empirical antifungals. Prophylaxis refers to use of antifungals without proven or suspected fungal infection but with risk factors for its development. Pre-emptive (diagnosis driven) approach means evidence of fungal infection, without definitive microbiological proof on the basis of surrogate biomarkers like 1-3 ß-D-glucan, mannan or anti-mannan antibodies, whereas empirical (fever-driven) approach refers to using antifungals in patients at risk for IFI, with signs and symptoms of infection, in the absence of microbiological evidence of infection.^457^

Among fungal pathogens, *Candida* spp. are the most commonly isolated microorganisms, currently being the fourth most commonly identified pathogens in nosocomial BSIs and the third most common pathogens isolated in ICU patients.^458^ Despite advances in antifungal therapy, the mortality associated with invasive candidiasis remains as high as 40%.^457^ In India, the incidence of *C. albicans* ranges from 34 to 45.6 % with an attributable mortality of 20–35.6%. The incidence of non-albicans *Candida* is on the rise with attributable mortality ranging from 23–52%, with higher mortality associated with *Candida krusei*.^459^ An observational study from Indian ICUs revealed an incidence of 6.5 cases per 1000 ICU admissions. There was a high prevalence of *C. tropicalis* (41.6%), and 46.6% isolates were susceptible to all antifungals. Fluconazole resistance was 5.2% for *C. albicans* while it was 2.6% for *Candida tropicalis*. Risk factors for invasive candidemia were found to be surgery especially abdominal surgery, central venous catheters, invasive mechanical ventilation, urinary catheterization, hemodialysis, and total parenteral nutrition.^164^

### What are the Risk Factors for Invasive Fungal Infections in ICU?

Risk factors for invasive fungal infections (IFIs) in ICU have been studied extensively. A large retrospective study in 301 surgical ICU patients found the risk factors for IFI to be peripheral and central intravenous catheters, bladder catheters, mechanical ventilation, lack of enteral nutrition and TPN.^460^ In a prospective study of 150 cardiothoracic ICU patients, risk factors for IFIs were prolonged mechanical ventilation (>10 days), hospital-acquired bacterial infection, cardiopulmonary bypass duration greater than 120 min, diabetes mellitus and high APACHE II score (> 30).^461^ A systematic review demonstrated that major surgery (OR–7.3), TPN (OR–3.8), fungal colonization with colonization index >0.5 (OR–19.1), hemodialysis (OR–3.8), acute renal failure (OR–4.2), severe sepsis, mechanical ventilation >3 days, diabetes (OR–2.8), APACHE 2 score > 16 (OR–1.03), cardiopulmonary bypass >120 min (OR–8.1), use of broad spectrum antibiotics (OR–3), red cell transfusion and central or peripheral venous catheters were significantly associated with IFIs.^462^

#### Evidence Statement

Risk factors for invasive fungal infections in non-neutropenic patients in ICU are surgery, total parenteral nutrition, renal replacement therapy, cardiopulmonary bypass >120 minutes, diabetes mellitus, central venous catheters, urinary catheters, *Candida* colonisation with colonization index >0.5, use of broad-spectrum antibiotics, acute renal failure, mechanical ventilation >3 days and APACHE II score >16.

### What is the Role of Empirical Antifungals in Non-neutropenic Patients in ICU?

The advantage of empirical antifungal treatment has already been established in high-risk patients such as cancer patients and solid organ transplant recipients in various studies.^463-465^ However, in non-neutropenic critically ill patients, the definitive evidence for the efficacy of untargeted treatment in terms of prevention of IFIs or mortality benefit has been equivocal. Moreover, studies have shown the potential detrimental effects of the injudicious use of antifungal agents in the form of the emergence of drug resistance, side effects, and financial costs.^466-468^ Several randomized controlled trials have compared empirical antifungals to placebo in non- neutropenic critically ill patients.^469-474^ In an RCT including post-surgery patients, fluconazole reduced the occurrence of candidemia (5.8% in fluconazole *vs*. 16% in placebo) through the mortality rates were similar.^469^ Similarly, use of caspofungin was also associated with a trend towards decreased IFI without any difference in mortality or length of hospital stay.^471^ A systematic review demonstrated that although empirical antifungals in non-neutropenic patients in ICU reduced the incidence of subsequent IFI, it had no impact on mortality.^474^ In a randomized controlled trial involving 260 mechanically ventilated patients with *Candida* colonization, empirical micafungin administration reduced the rate of subsequent proven IFI (12% *vs*. 3%; p = 0.008) without any significant mortality benefit.^473^

#### Evidence Statement

Empirical antifungals for non-neutropenic patients in ICU routinely has not been associated with a decrease in mortality or hospital length of stay.

Empirical antifungals in patients at high risk for invasive fungal infections in ICU has been shown to reduce the incidence of subsequent proven invasive fungal infections.

#### Recommendations

- We do not recommend the routine use of empirical antifungals in non-neutropenic patients in ICU (1A).
- Empirical antifungals may be considered in critically ill patients with a high risk of invasive fungal infections to reduce the incidence of subsequent invasive fungal infections (1B).

### What is the Antifungal Agent of Choice and

### Duration of Empirical Therapy in Non-neutropenic Patients in ICU?

The options for antifungal therapy include fluconazole, amphotericin-B, and echinocandins. In a systematic review, empirical use of fluconazole and caspofungin reduced rates of subsequent IFI while micafungin, nystatin, and amphotericin-B did not.^474^ No direct comparative data of efficacy of different antifungals for empirical therapy in non-neutropenic patients in ICU is available. Indian studies have shown an increased prevalence of non-albicans *Candida* with high rates of fluconazole resistance in the range of 5 to 7%.^475^ Regarding duration of empirical antifungal therapy, and there are no studies directly comparing the different duration of empirical antifungal therapy. Most of the studies have used at least 2 weeks therapy.^474^

#### Evidence Statement

Fluconazole and caspofungin are useful as empirical antifungal therapy in non-neutropenic ICU patients at high risk of invasive fungal infection. In India, the rate of fluconazole resistance is up to 7%, especially in non-albicans *candida* species.

#### Recommendations

- We recommend fluconazole or caspofungin as preferred empirical antifungal agents in non-neutropenic ICU patients at risk for invasive fungal infection (1A).
- Caspofungin may be preferred in areas with high prevalence of fluconazole resistance (1B).
- Micafungin or anidulafungin may be used as alternative agents (3A).
- Recommended duration of empirical antifungal therapy is 2 weeks (3A).

### Antibiotic Stewardship

Antibiotic stewardship program is defined as “coordinated interventions designed to improve and measure the appropriate use of antibiotic agents by promoting the selection of the optimal drug regimen including dosing, duration of therapy, and route of administration.”^476^ An efficient antibiotic stewardship program results in optimum clinical outcomes while reducing adverse effects of unnecessary antibiotic use. Every additional 10 days of antibiotic therapy conferred a 3% increased risk of an adverse drug event. These adverse effects include the emergence of antibiotic resistance, *Clostridium difficile* infections and drug toxicity and occurs in 20% of patients.^477^A structured antibiotic stewardship program requires a multidisciplinary approach. Core elements of antibiotic stewardship program include committed leadership, accountability, expertise in drugs, action, tracking drug resistance patterns, regular reporting and education to clinicians about optimal prescribing.^478^

### Does Antibiotic Stewardship Improve Patient Outcome in ICU?

Antibiotic stewardship programs reduced the duration of antibiotic treatment (1.95 days; 95% CI 2.22–1.67) and duration of hospital stay (1.12 days, 95% CI 0.7–1.54) without any significant difference in mortality in a recent systematic review.^478^ In a recent meta-analysis, there was reduced mortality with guideline directed empirical therapy (RR 0.65, 95% CI 0.54–0.80, p < 0.0001) and antibiotic de-escalation (RR 0.44, 0.30–0.66).^479^ Mortality benefit has also been reported in another systematic review (RR 0.68, 95% CI 0.52–0.88).^480^ However, a single non blinded randomized study showed a significantly higher rate of superinfection with the de-escalation of antibiotics as compared to continuation of empirical therapy (27% *vs*. 11%; p = 0.03).^481^

#### Evidence Statement

Antibiotic stewardship programs in hospitalized patients are associated with a reduction in a number of antibiotic days, duration of hospital stay and all-cause mortality.

#### Recommendations

- All hospitals should have an antibiotic stewardship program including the intensive care units (2A).

### What are the Essential Strategies of Antibiotic Stewardship in an ICU Setting?

Prospective audit-feedback and preauthorization are commonly used strategies of antibiotic stewardship.^482-484^ In prospective audit and feedback, treating clinicians are provided recommendations regarding the appropriateness of antibiotics used. Advantages of this strategy include avoidance of delay in antibiotic administration (as the physician is engaged after prescription of antibiotics). Limitations of this strategy include partial compliance (due to voluntary participation of physicians), resource intensive nature, and a longer lag period for visible benefits to become apparent. Prospective audit and feedback strategy resulted in a reduction in the utilization of antibiotics and significant cost reduction.^485,486^ In a systematic review, enabling strategies including feedback resulted in greater efficacy of stewardship interventions.^482^ Pre-authorisation, another strategy of antibiotic stewardship, requires approval by the concerned authority before starting antibiotics.^483^ This affects the use of restricted antibiotics only and may result in a potential delay in antibiotic initiation. Without feedback, this may also result in increased use of other antibiotics and hence lead to the selection of different resistance patterns. However, it provides immediate results in terms of reduced antibiotic usage. Other potential drawbacks include the development of negative professional culture because of a breakdown in communication between infectious disease specialists and clinical teams.^482^ Enabling and restrictive strategies have been compared in a quasi-experimental crossover trial using days of antibiotic therapy in both strategies.^487^ In this study involving 2,686 patients in pre-prescription authorization (PPA) group and 2693 patients in post-prescription review with feedback (PPRF) group, initially antibiotic days of treatment (DOT) remained relatively unchanged in the PPA arm. When changed to the PPRF arm, antibiotic use decreased (-2.45 DOT per 1000 patient-days (PD)] hence concluding that PPRF may have more impact on decreasing days of antibiotic therapy.

#### Evidence Statement

Antibiotic stewardship requires a multidisciplinary approach with integration of infectious disease physician, a microbiologist with logistic and financial support from hospital administration. Both enablement and restrictive strategies are useful in improving adherence to antibiotic stewardship programs. Restrictive strategies give immediate results. Enablement practices are more resource intensive. Most studies have used a combination of both the methods and have shown additive effects. Providing feedback to the treating team improves adherence. A single RCT has shown that a restrictive strategy alone may cause a delay in the initiation of antibiotics.

#### Recommendations

- Prospective audit of antibiotic use and/or preauthorization (if feasible) along with feedback to the treating team is recommended as part of an antibiotic stewardship program (1A).

### What is the Role of Antibiotic Cycling, Intravenous to Oral switch and De-escalation in the ICU?

Antibiotic cycling refers to withdrawing a specific antibiotic or an antibiotic class from use for a definite period and substituting with another antibiotic or antibiotic class having a similar spectrum of activity.^483^ This is postulated to induce different resistance mechanisms in the microorganisms and hence prevent or reverse the development of antibiotic resistance. There is no compelling evidence on the benefit of antibiotic cycling in terms of clinical endpoints. Several prospective before and after studies without control groups have demonstrated a reduction in the incidence of ventilator-associated pneumonia (6.7% with antibiotic cycling as against 11.6% before the intervention)^489^ as well as a reduction in colonization.^489-491^ A newer prospective cohort study^492^ comparing antibiotic mixing, and antibiotic cycling found no significant differences in infection rates (16.6% and 14.5%, OR 0.9), infection due to target microorganisms (5.9% and 5.2%, OR 0.9), hospital length of stay (median 5 days for both groups) or in-hospital mortality (13.9% and 14.3%, OR 1.03).

#### Evidence Statement

Antibiotic cycling in the intensive care unit has not been adequately studied in randomized controlled trials. Non-randomized studies show significant heterogeneity in terms of site of study, a method of cycling and confounders like simultaneous infection control measures being employed. Evidence of benefit of antibiotic cycling is lacking, with few studies demonstrating a reduction in colonization though mortality and length of hospital stay remain unchanged.

#### Recommendations

- Antibiotic cycling should not be used as a method of the antibiotic stewardship program (2A).

### Scheduled Intravenous to Oral Switch

Timely switch from intravenous to oral antibiotics has been shown to reduce the cost of health care and length of hospital stay.^493-498^ In case of antibiotics with the availability of equivalent oral formulations, the scheduled switch is easier than in case of broad-spectrum antibiotics without oral formulations or precise like piperacillin-tazobactam or meropenem. A multicenter randomized controlled trial done in CAP which evaluated scheduled switch to oral antibiotics after 2 days of intravenous antibiotics found similar cure rates, survival or resolution of chest radiology with a significantly lower total cost of care (2953$ and 5002$, p < 0.05).^497^ Oosterheert et al.^496^ also found similar results when comparing scheduled switch on day 3 and day 7 with similar cure rates and mortality rates in both groups but with significantly reduced duration of intravenous antibiotics and hospital stay, with differences of 3.4 days and 1.9 days respectively.

#### Evidence Statement

Early intravenous to the oral transition of antibiotics reduce hospital length of stay and cost of care. There is no increase in mortality or other adverse events when this is done after assessing as to which patients can be safely transitioned to oral therapy.

#### Recommendations

- Antibiotic stewardship programs should implement strategies to improve the timely transition from parenteral to oral antibiotic therapy (2A).

### De-escalation in Intensive Care Unit

Antibiotic de-escalation refers to a strategy of switching from broad-spectrum antimicrobials to a narrower spectrum of antimicrobials. It is recommended to reduce the emergence of multidrug-resistant bacteria as well as costs of health care. In a multicenter randomized controlled trial, de-escalation was associated with a longer ICU stay but similar in-hospital mortality in severe sepsis.^481^ In a recent meta-analysis of 9 studies involving 1873 patients with septic shock, de-escalation of antibiotics was associated with a trend towards reduced mortality (RR 0.74, 95% CI 0.54–1.03).^499^ In another systematic review, de-escalation was associated with lower mortality (RR 0.68; 95% CI 0.52–0.88).^480^

#### Evidence Statement

Pooled results from observational studies in an ICU setting do not show any increase in mortality with antibiotic de-escalation while significantly reducing antibiotic exposure days and ICU length of stay.

#### Recommendations

- Antibiotic de-escalation in the ICU is recommended as part of an antibiotic stewardship program (2A).

### What is the Role of Procalcitonin in Antibiotic De-escalation ICU?

Procalcitonin is a 116 amino acid precursor to calcitonin. Normal serum or plasma levels of procalcitonin in healthy adults are <0.05 ng/mL. It can be produced by a variety of cell types in response to inflammatory stimuli, especially of bacterial origin. It does not usually rise significantly in response to viral or non-infectious inflammation and so has the potential to be used as a marker of bacterial infection. The levels in serum are quantified using immunoassay.^127^ Procalcitonin use to guide antibiotic therapy in sepsis in intensive care unit resulted in reduction in antibiotic days (MD–3.19 days, 95% CI –5.44- – –0.95) duration of hospital stay (MD–3.85 days, 95% CI -6.78–0.92) as well as a trend towards reduction in duration of ICU stay (MD–2.03 days, 95% CI -4.19–0.13 days).^500^ Procalcitonin-guided algorithm for antibiotic discontinuation (decrease by > 80% of peak value, or < 0.5 ng/mL) led to reduced antibiotic administration (between-group absolute difference 1·22, 0·65–1·78, p <0·0001), with significant mortality benefit (20% *vs*. 25%; between-group absolute difference 5·4%, 95% CI 1·2–9·5, p = 0·0122).^501^ In a recent Cochrane meta-analysis involving 26 trials, procalcitonin utilization for antibiotic discontinuation was associated with reduced mortality (adjusted OR 0.83, 95% CI 0.70 to 0.99, p = 0.037).^84^

#### Evidence Statement

Implementation of antibiotic de-escalation algorithm based on serial procalcitonin measurements has been shown to reduce mortality, length of ICU stay, the total duration of antibiotic days and health care costs.

#### Recommendations

- Procalcitonin based algorithms may be used for antibiotic de-escalation (1A).

### Author Details

**Other Contributors:** Avneet Garg, Dilip Dubey, Hariharan Iyer, Jugendra Singh, Prashuram Bista, Sachin Doddamani, Sandip Agarwal, Shiba Kalyan Biswal, Shweta Bansal, Swapnil Mehta, Sryma PB, Tajammul Hussain Shah, Tejas Suri

*For the Expert Committee to formulate National Guidelines for Antibiotic Prescription in ICU

*Expert Committee: Arti Kapil, Pawan Tiwari, Saurabh Mittal, Dhruva Chaudhary, JC Suri, MK Daga, Yash Zaveri, Suresh Rama Subban, Seema Sood, RK Mani, Narendra Rungta, Anirban Chaudhry, Rajesh Pandey, Neetu Jain, Arvind Baronia, Jaya Kumar, Gyanendra Agarwal, Camilla Rodrigues, BK Rao, Deepak Govil, Sachin Gupta, Ashit Hegde, Pramod Garg, Sandeep Mahajan, Chand Wattal, Rajesh Chawla, Anjan Trikha, Prakash Shastri, Anil Gurnani, Rajesh Mishra, Rohit Bhatia, GC Khilnani, Kapil Zirpe, Vijay Hadda, Anant Mohan, Atul Kulkarni, Karan Madan, Yatin Mehta, Subhal Dixit, Randeep Guleria, Pradeep Bhattacharya
